# Huckleberry Habitat and Its Influence on Two Small Populations of Grizzly Bears (*Ursus arctos*)

**DOI:** 10.1002/ece3.72905

**Published:** 2026-01-19

**Authors:** Justin E. Teisberg, Wayne F. Kasworm, Michael F. Proctor, Thomas G. Radandt, Jennifer K. Fortin‐Noreus, Hilary S. Cooley

**Affiliations:** ^1^ US Fish and Wildlife Service Libby Montana USA; ^2^ Birchdale Ecological Ltd Kaslo British Columbia Canada; ^3^ US Fish and Wildlife Service University of Montana Missoula Montana USA

**Keywords:** diet, energetics, grizzly bear, habitat, huckleberry, resource selection function, *Ursus arctos*

## Abstract

Huckleberries (*Vaccinium* spp.) are a nutritionally important food to grizzly bears ( *Ursus arctos* ) in interior, western North America. They provide sugar‐rich calories in late summer and fall prior to denning. We developed a resource selection function of high‐quality huckleberry habitat important to Cabinet‐Yaak and Selkirk female grizzly bears using field‐verified huckleberry foraging radiolocations acquired during prime months of huckleberry fruiting (July 15–September 15, 2010–2019, *n* = 22). Logistic regression analysis identified a model with 12 significant variables in predicting huckleberry habitat important to female grizzly bears (Somers' D = 0.729; K‐S statistic = 0.570, *p* < 0.00001). Most influential variables (*p* < 0.00001; positive [+] or negative [−] relationship) include canopy closure (−), moisture deficit (−), time since last wildfire (−), solar radiation (+), snow water equivalent (−), and growing degree days above 5°C (−). On average, 28% of an adult female annual home range includes predicted huckleberry habitat ($\bar{x}$ = 61 ± 6.4 [SE] square kilometers). Seasonal ranges of females overlap extensively within predicted huckleberry habitat, and degree of overlap trends with quality of habitat. Mothers and daughters display similar selection patterns for predicted huckleberry habitat, suggesting huckleberries are an important component of dispersal patterns, range expansion, and connectivity (via linkage areas) to other populations. Model values were significantly associated with long‐term berry production, allowing estimates of amount of this food resource available on the landscape. Using energetic predictions of huckleberry foraging in these areas, we find that a smaller average body size of Cabinet‐Yaak and Selkirk adult females ($\bar{x}$ = 94 kg lean body mass) lessens the energetic constraints of a huckleberry‐dominant diet and may be a direct outcome of huckleberries being a primary food resource for these populations.

## Graphical abstract

We modeled habitat of an important seasonal food item for two Threatened (United States Endangered Species Act) populations of grizzly bears. Our findings suggest that huckleberry habitat is not limiting but does have the potential to influence population density, dispersal/range expansion, energetics, and body size. Considerations of habitat can add value and insight into population linkage planning (i.e., “wildlife connectivity zones”) and population density expectations.

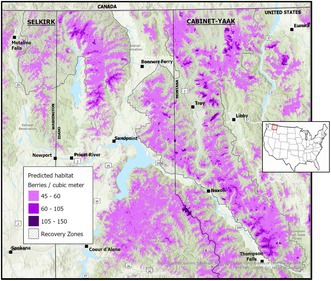

## 1 Introduction

Grizzly bears ( *Ursus arctos* ) in the Cabinet‐Yaak and Selkirk Ecosystems are listed as federally Threatened under the United States Endangered Species Act and are managed within and around two designated Recovery Zones within the states of Idaho, Montana, and Washington (USFWS 1993). Both populations contain a small number (≤ 100 each) of bears and are inherently vulnerable to stochastic effects, human‐caused mortality, habitat modification, and habitat fragmentation. Long‐term persistence and recovery of these grizzly bear populations will require limited human‐caused mortality, adequate genetic and/or demographic interchange of individuals among populations, as well as sufficient cub production and recruitment (Wakkinen and Kasworm 2004; Proctor, Servheen, et al. 2004).

Many studies have documented bottom‐up factors that influence grizzly bear populations (McClelland et al. 2021; McLellan 2011; Nielsen et al. 2016; Proctor et al. 2023). The extent to which certain foods, and their associated habitat, may influence or assist recovery of these small populations is less understood. Berries, specifically those within genus *Vaccinium* (globe huckleberry [ *Vaccinium globulare* ], black huckleberry [ *Vaccinium membranaceum* ], dwarf huckleberry [ *Vaccinium caespitosum* ], and whortleberry [ *Vaccinium scoparium* ]), are a key food item for Cabinet‐Yaak and Selkirk grizzly bears in summer and early fall because they provide sugar‐rich calories for maintenance and fat accumulation. In other nearby populations, the fruit is considered a critical food to grizzly bears (McLellan 2015; McLellan and Hovey 1995). In our local ecosystems, upwards of 70% of dry matter consumed consists of berries (primarily huckleberry) in mid‐summer based on scat analysis and hair and blood isotope signatures (Kasworm et al. 2024b, Teisberg, personal communication). Higher annual indices of berry production are associated with lower human‐caused mortality in these two populations (Kasworm et al. 2024a). Radio‐location data also suggest bears are largely in more remote habitat as they forage for huckleberries (Kasworm et al. 2024a). Obtaining a greater understanding of availability and spatial extent of this important food may offer insight into habitat capacity and its relative effect on density and biological carrying capacity (Lyons et al. 2018; McClelland et al. 2021; Nielsen et al. 2016). Regionally, productive huckleberry shrubfields have been described and modeled and, in general, are often found under open timber, rocky parks, in old burns, in cutting units, or high‐elevation open alpine—areas with relatively open canopy, often associated with disturbance in the past 10–50 years (Minore 1984; Barney 1999; Barney et al. 2006; Nielsen and Nielsen 2010; Holden et al. 2012; Roberts et al. 2014; Proctor et al. 2023; Shores et al. 2019; Hobby and Keefer 2010; Souliere et al. 2020). Additionally, berry habitat can be a result of human prescription (e.g., harvest and/or prescribed burn) implemented by land management entities such as the U.S. Forest Service, state natural resource departments, and private timber corporations.

High‐quality foods, such as berries, influence landscape distribution, range expansion, and connectivity of grizzly bear populations. As a necessary requirement, food resources influence the size and shape of the established range of a bear (Dahle and Swenson 2003; Edwards et al. 2013). A population's spatial extent is the composite range of successive generations of female bears, with expansion driven by range selection of dispersing, subadult females (McLellan and Hovey 2001; Proctor, McLellan, et al. 2004; Zedrosser et al. 2007). Often, grizzly bear ranges abut or are divided by highways or areas of dense human development, such as with the Selkirk and Cabinet‐Yaak populations. Movement and gene flow between small, fragmented populations occur within functional linkages (i.e., “connectivity zones”), those areas that facilitate connections and movement of animals (USFWS 1993; Proctor et al. 2005, 2012, 2015). Spatial modeling and identification of these linkage areas guides the placement of effective crossing structures and effective land acquisitions. The inclusion of foods and foraging habitat within connectivity modeling can identify nutritionally‐attractive areas, those that are potentially nutritional sinks, where food abundance may conflict with human presence and pose serious constraints to connectivity (Lamb et al. 2016).

For grizzly bears, nutritionally important foods are calorically dense and have balanced macronutrient content, thereby directly influencing cub production and recruitment through sufficient mass gain (Erlenbach et al. 2014; Hertel et al. 2018; Hilderbrand et al. 1999, 2019; Mangipane et al. 2020; McLellan 2011). However, calorie acquisition and ultimate mass gain are a complex balance between energy intake and energetic demand, depending on many factors in concert (e.g., nutritional quality of the food resource, foraging efficiencies, resource availability and distribution, intra‐ and inter‐specific competition). Even more, caloric demand depends on yet another long list of factors, including body size, movement rates, and reproductive status of the animal (Kleiber 1932). While nutritionally valuable, energetic constraints limit the value of berry foraging, such that smaller bears are more likely to meet their energetic requirements at maximum ingestion rates, whereas larger bears cannot (Felicetti et al. 2003; McLellan 2011; Robbins et al. 2007; Rode et al. 2001; Rode and Robbins 2000; Welch et al. 1997). Grizzly bears exhibit a high degree of phenotypic plasticity in terms of their morphometry and body size (Cameron et al. 2020; McDonough and Christ 2012; Zedrosser et al. 2006). Body size is largely determined by nutrition during dependent, subadult, and early adult years for bears, when growth plates are open and body structure is built (Kingsley et al. 1988). Similar to other nearby populations, female grizzly bears within the Cabinet‐Yaak and Selkirk ecosystems are small‐sized, interior grizzly bears, with one of the lowest average lean body mass measures (~115 kg) of North American populations (McLellan 2011; Hilderbrand et al. 2017; Kasworm et al. 2024b). This physiologic outcome may be the result of berries being one of a few important foods for Cabinet‐Yaak and Selkirk grizzly bears.

Resource selection functions (RSFs) provide information specific to species spatial distribution, producing values proportional to the probability of use of a resource unit (Boyce and McDonald 1999; Manly et al. 2007). Researchers typically parameterize RSFs using logistic regression, where values of environmental variables at known locations of an animal are regressed against values from points assumed to be available to the animal. This approach allows researchers to estimate selection above or below what would be expected, producing maps and spatial layers for further analyses and understanding. Within this framework, it is important that investigators carefully consider and explicitly define spatial and temporal scale, such that the use‐available location data matches the intent of the modeling exercise (Boyce 2006; Ciarniello et al. 2007; Denny et al. 2018; Johnson 1980). In our case, we developed a fine‐scale RSF that predicts berry foraging habitat important to grizzly bears, focusing first on 4 order selection (i.e., specific locations of the resource, in this case huckleberries) to identify habitat patches within grizzly bear home ranges (Johnson 1980). Considering this scale and intent, we: (1) limited model development to locations confirmed by ground‐truthing; (2) conservatively constrained our definition of available range, based on phenology and ripeness of the resource; and (3) only used abiotic, vegetative, physiographic, edaphic, and disturbance features to predict where huckleberry habitat occurs, based on grizzly bear use. We also specifically modeled female grizzly bear foraging habitat, as female range and food selection has direct influence on recruitment to adulthood, cub production, overall fitness, and ultimately, population trajectory.

Using the developed model, we: (1) assessed what proportion of female home ranges typically includes huckleberry habitat, and whether it varies among females; (2) estimated if and to what degree seasonal overlap among females is associated with quality of berry habitat (via probability of use); (3) compared huckleberry habitat use by adult and subadult females, as well as mothers and their dispersal‐aged daughters; and (4) estimated huckleberry habitat in terms of potential calories available, secondarily applying foraging energetics to assess consequences of body size for grizzly bears with diets predominantly composed of huckleberry.

## 2 Methods

### 2.1 Study Area

The Cabinet‐Yaak ecosystem (48.4° N 115.7° W) includes habitat and occupied grizzly bear range within northwest Montana, northern Idaho, and southeastern British Columbia (BC). The Kootenai River and associated highway separate female distribution into two centers, the Cabinet Mountains to the south and Yaak River and Purcell Mountains to the north. Much of the Cabinet‐Yaak Recovery Area is comprised of public land administered by the Kootenai, Lolo, and Idaho Panhandle National Forests. Private, small‐parcel, residential ownership exists mainly within major river valleys, which has the potential to fragment grizzly bear habitat and increase human‐bear conflicts between and within the two Recovery Areas.

The Selkirk ecosystem (49.0° N 116.8° W) encompasses habitat within the Selkirk Mountains of northeastern Washington, northern Idaho, and southern BC. Female grizzly bear range in the Selkirks is largely defined by secure habitat with lower road densities between the major drainages of the Kootenai (including Kootenay Lake, BC), Pend Oreille, and Columbia Rivers, and associated highways. Grizzly bear distribution straddles the US‐Canada border. Land ownership in the U.S. portion of the Recovery Area is mainly public (federal or state lands), administered by the Idaho Panhandle and Colville National Forests as well as the Idaho Department of Lands.

The Cabinet‐Yaak‐Selkirk landscape experiences a mix of temperate Pacific maritime and continental climates, with increased maritime influence in more westerly extents. Climate is generally characterized by short, warm summers and long, wet winters. The spring months of April to June include much rainfall, while the summer months of July and August are often quite dry. Most winter precipitation is in the form of snow.

Huckleberries are a deciduous shrub with medium height (0.1–2 m), with moderate to low shade tolerance (Hobby and Keefer 2010). They reproduce via underground rhizomes, more rarely via seed (Minore 1984). Huckleberries fruit abundantly only under ideal conditions. Productive plants are often found in well‐drained, nitrogen‐poor soils with pH ranging from 5.0 to 5.5, typically over neutral to acidic bedrock (Barney et al. 2006; Minore 1984). Plants are susceptible to early melting of snow and late‐frost damage to stems, leaves, and flowers. Timing of precipitation can also influence activity of pollinators, yet summer droughts can lead to failure of the berry crop (Barney 1999). Huckleberry shrubfields are often found under open timber canopies adjacent to graminoid parks, in old burns, in timber harvest units, and intermixed with beargrass ( *Xerophyllum tenax* ). These often mixed‐species shrubfields are partially a result of wildfires that occurred in 1910 and 1929 and more recent stand replacing fires, though some are the result of silvicultural practices. In recent decades, several stand altering fires have occurred across the study area. In recent years, the Kootenai and Idaho Panhandle National Forests have implemented prescribed fire and other forest management prescriptions to promote grizzly bear foraging habitat in the form of more open‐canopy berry shrubfields. Some private entities with larger tracts of land (i.e., timber corporations) have also attempted to promote wildlife habitat via similar forest management prescriptions.

### 2.2 Model Development

#### 2.2.1 Capture and Collaring

Capture and handling of bears followed an approved Animal Use Protocol through the University of Montana, Missoula, MT (061‐14CSCFC111714 and 040‐20HCCFC‐092420). Bears were captured with foot‐hold snares or culvert traps following the techniques described by Johnson and Pelton (1980) and Jonkel (1993). Bears were typically immobilized with a dissociative tranquilizer (Telazol; tiletamine and zolazepam) mixed with an alpha‐2 agonist (xylazine or dexmedetomidine) (Radandt 2009; Teisberg et al. 2014). Yohimbine and Atipamezole were the primary antagonists for xylazine and dexmedetomidine. Drugs were administered intramuscularly with a jab stick, blowgun, modified air pistol, or cartridge‐powered dart gun. Immobilized bears were measured, weighed, and a first premolar tooth was extracted for age determination (Stoneberg and Jonkel 1966). Immobilized bears were given oxygen at a rate of 2–3 L/min. Recovering bears were dosed with diazepam.

All grizzly bears were fitted with Global Positioning System (GPS) radio collars. Collars were manufactured by Telonics (Mesa, AZ). To prevent permanent attachment, a slow‐rotting cotton webbing spacer was the backup for a timed mechanical drop‐off device placed in the collars so that they would drop off in 1–3 years (Hellgren et al. 1988). Collars were programmed to attempt GPS location acquisitions every 2 or 3 h, resulting in a maximum of 12 locations per day per bear.

#### 2.2.2 Location Selection and Ground‐Truthing

We used subadult and adult female grizzly bear GPS collar location data from the Cabinet‐Yaak and Selkirk ecosystems (12 and 10 bears, respectively). First, we selected all 3‐dimensional fix GPS locations (± 50‐m accuracy) between July 15 and September 15, a range of dates known to fall within huckleberry fruiting season in the Cabinet‐Yaak and Selkirk ecosystems. We also limited location data to the most recent decade (2010–2019) to minimize the influence of succession on our ability to infer huckleberry site feeding selection and tighten our temporal timeframe of modeling. For each female, we randomly selected a subset of seasonal GPS locations for physical visit and ground‐truthing.

At ground‐truthing locations, field crews collected data specific to presence and abundance of plants and fruits for the two predominant species of huckleberry (globe huckleberry: *Vaccinium globulare* ; and black huckleberry: *Vaccinium membranaceum* ), as well as other fruiting species thought to be important food for grizzly bears that occur to a lesser extent (*Vaccinium scoparium, Amalanchier alnifolia, Shepherdia canadensis, Sorbus scopulina, Ribes* spp.). It should be noted that *V. globulare* and *V. membranaceum* are nearly indistinguishable (Hobby and Keefer 2010; Richards and Alexander 2006). For the sake of simplicity, we distinguish all research outcomes herein pertaining to taxon *Vaccinium globulare* (hereafter “huckleberries”).

Sites were visited during the median 4 weeks of huckleberry fruiting season (month of August). Due to collar and navigational device GPS location inaccuracies, observers first determined a practical site center, within a 20‐m radius of the device‐specified location, considering foods present and bear sign observed. At each site, observers collected measurements at five microplots, each 1 square meter in area, one at site center and four others 10 m from site center in the 4 cardinal directions. Within the microplots, observers evaluated plant species, percent coverage, presence of fruit, and counts of fruits in the plot. Fruit counts were summarized into categorical bins, established from the observed distribution of counts at historic transect locations in the Cabinet‐Yaak and Selkirks, 1989–2021 (Kasworm et al. 2024a). Observers also gave an independent assessment of whether the location provided berry forage for the located grizzly bear (e.g., “yes”, “no”, or “possible” foraging site). To frame their expert opinion, biologists noted and considered sign of feeding, existence and size of foraging patch, quality of foraging patch, past and recent disturbance and/or successional processes, evidence to suggest bedding at or travel through a site, and other ecological context. All observers were trained field biologists with years of grizzly bear community and ecology field work experience and knowledge.

#### 2.2.3 Modeling Process

We first established a model training dataset that included “use” locations (i.e., ground‐truthed huckleberry foraging sites of female grizzly bears) and “available” locations. This training dataset was used to develop a resource selection function (RSF; Manly et al. 2007). Our goal was to model landscape‐level similarity among female grizzly bears in their 4 order selection of berry habitat. As such, we combined all location data for analyses (i.e., unconditional logistic regression). We conservatively defined use locations as sites where both expert opinion and empirical measurements of huckleberries aligned, via logistic regression analysis (see Supporting Information). Available locations were randomly selected points within a composite, seasonal (July 15—September 15) minimum convex polygon of all sampled females within each population, at a rate 10 times that of use points (Figure S1) (Proctor et al. 2023). We chose this approach for two reasons: (1) Cabinets, Yaak, and Selkirks are functionally separate populations with regard to female movements, and we could not justify using points between populations; and (2) seasonal ranges are a more reasonable depiction of what is available to the bear during the berry fruiting season (Kasworm et al. 2024a). This approach also offered cross‐ecosystem opportunity to evaluate connectivity, through assessment of common habitat between study areas.

We collected spatial layers for 28 environmental variables hypothesized to be important to productive huckleberry presence and productivity (Table 1; Minore 1984; Barney 1999; Barney et al. 2006; Nielsen and Nielsen 2010; Holden et al. 2012; Roberts et al. 2014; Proctor et al. 2023; Shores et al. 2019; Hobby and Keefer 2010; Souliere et al. 2020). Most relationships were intuitive based on ideal environmental conditions (see Study Area), but some were more nuanced. For instance, we predicted that long‐term moisture was important for vegetative growth, yet short‐term precipitation was less so, and likely having a different coefficient, due to its impact on pollinator activity. We extracted values for each variable at each point in our training dataset. We created a model training dataset by randomly selecting ~80% of females from the dataset (Boyce et al. 2002). We used a forward selection process, starting with a simple model that included one variable (tree canopy openness) known to predict huckleberry shrubfields in other interior North America grizzly bear habitat (Hobby and Keefer 2010; Proctor et al. 2023; Shores et al. 2019). We then added a variable if (1) it was a significant predictor (*p* < 0.05) and (2) the addition resulted in a model with lower Akaike Information Criterion (ΔAICc > 2), to avoid including uninformative parameters (Leroux 2019). The process was repeated with remaining variables and their marginal results. We assessed presence of multicollinearity between variables and did not include those that had correlation coefficients of > 0.8 or < 0.8. Our primary intent was to develop a predictive model. All model building and assessments of model fit were executed with program STATISTICA (Dell Inc. 2015). We then computed and plotted relative selection strength for each variable within the final model, which allowed for interpretation of predicted selection over the range of values, holding all other variables at their mean values (Avgar et al. 2017).

**TABLE 1:** Environmental variables (*n* = 28) included in model development. All variables were expected to have some influence on presence and productivity of huckleberry plants in the Cabinet‐Yaak‐Selkirk study area. A short explanation, data source, expected relationship, and citation of evidence are included. Some variables did not make final model selection.

Variable	Source	Expectation	Evidence in literature
Tree canopy closure (2016)	Landfire (USFS)	−	1, 2, 3, 4, 5
Hargreaves climatic moisture deficit	ClimateNA (BC)	−	1
Coarse fragments in soils	WSS NRCS (USDA)	−	1, 6
Compound topographic index	DEM (USGS)	−	1, 7
Degree‐days above 5°C	DAYMET (NASA)	−	8
Frost free period	DAYMET (NASA)	−	1, 8
Time since last fire (years, from 2015)	Landfire (USFS)	−	1, 5, 9
Mean annual temperature	DAYMET (NASA)	−	8
Mean coldest month temperature (°C)	DAYMET (NASA)	−	8
Mean annual precipitation (2010–2015)	DAYMET (NASA)	+	1
Mean annual precipitation (1980–2015)	DAYMET (NASA)	+	1
Mean annual summer (May to Sept) precipitation	DAYMET (NASA)	+	1
Mean annual solar radiation (2010–2015)	DAYMET (NASA)	+	1
Mean annual solar radiation (1980–2015)	DAYMET (NASA)	+	1
Mean annual snow water equivalent (2010–2015)	DAYMET (NASA)	+	1, 8
Mean annual snow water equivalent (1980–2015)	DAYMET (NASA)	+	1, 8
Mean warmest month temperature (°C)	DAYMET (NASA)	+	8
Number of frost‐free days	DAYMET (NASA)	+	1, 8
Percent organic carbon in soil	WSS NRCS (USDA)	+	6
Soil ph, dissolved using water	WSS NRCS (USDA)	−	6, 10
Summer heat moisture index	ClimateNA (BC)	+	1
Slope (°)	DEM (USGS)	−	1, 7
Average temperature—winter	DAYMET (NASA)	−	8
Percent clay in soil	WSS NRCS (USDA)	−	6
Maximum temperature—summer	DAYMET (NASA)	−	1, 8
Minimum temperature—spring	DAYMET (NASA)	−	8
Minimum temperature—winter	DAYMET (NASA)	+	1
Percent sand in soil	WSS NRCS (USDA)	+	6

*Note:* (1) Proctor et al. (2023), (2) Shores et al. (2019), (3) Hobby and Keefer (2010), (4) Minore (1984), (5) Souliere et al. (2020), (6) Barney et al. (2006), (7) Roberts et al. (2014), (8) Holden et al. (2012), (9) Nielsen and Nielsen (2010), (10) Barney (1999).

#### 2.2.4 Assessment of Model Fit

We computed a list of model fit statistics to assess (1) model classification performance and (2) best model value cutoffs to define quality huckleberry habitat (Table S1). Receiver operating characteristic (ROC) curves and area under the curve (AUC) scores were developed and computed within STATISTICA (Dell, 2015). We performed 100 replications of model development, testing model fit against the withheld ~20% of females in the full dataset (Boyce et al. 2002). This approximate ratio is the result of maintaining independence between training and test datasets; no bear had locations in both training and test datasets during one comparative replication. ROC curve and AUC interpretations are as in Hanley and McNeil (1982). Because no model is a perfect estimator, each model value cutoff carries a likelihood of correctly predicting whether a raster cell contains productive huckleberry habitat; in other words, we accept both a probability and inherent uncertainty as to whether the cell contains huckleberry habitat. After selecting relevant likelihood odds‐ratio (LOR) model cutoffs, we chose to define the modeled huckleberry landscape in terms of an explicit likelihood that a grid cell contains huckleberry habitat, rather than a binary “yes” or “no” based on a single cutoff value. This was computed from a significant regression of binned model values and empirical site visit data, summarized as huckleberry “presence” or “occupancy” rates (Figure S2). This logic further translated into estimates of absolute area in which we chose to not assume the aggregate of a cell contained all or no huckleberry habitat. Other studies have estimated absolute area of habitat as the aggregate of total area of all cells with model values above or within a certain threshold range (Sorensen et al. 2015). Instead, we interpreted values as an “occupancy probability”, or percentage area of the grid cell containing productive huckleberry, to estimate absolute abundance of habitat (Guillera‐Arroita et al. 2015). In our region, productive huckleberry shrubs often exist as small, separated patches, not as vast contiguous shrubfields. As such, we find these methods better estimate true amounts of huckleberry habitat on the landscape.

### 2.3 Predicted Habitat Within Female Ranges

We estimated the annual and berry seasonal (July 15—September 15) range for all adult and subadult females 2010–2019, by means of a minimum convex polygon (MCP) created in a GIS (ArcGIS Pro 3.3.6). Subadult females were defined as nonreproductive, independent, dispersal‐aged (typically 3 to 4 years old), while adults were defined as 5 years or older. We then summed the total predicted area of berry habitat within each home range and calculated means and proportions from these data. We assessed differences between age classes of females as well as differences in habitat composition between genetically confirmed dyads (i.e., adult females and their subadult, dispersal‐aged daughters). Statistical differences were assessed with a one‐sample *t*‐test.

### 2.4 Berry Production and Energetics

Long‐term huckleberry fruit production was estimated at independent sites across the Cabinet‐Yaak and Selkirks, using methods similar to Kendall (1986). Transect line origins were marked and a specific azimuth was followed from the origin through homogenous habitat. At 0.5 m intervals, a 0.04 m^2^ frame (2 × 2 decimeter) was placed on the ground or held over shrubs. Site locations were not associated with the female grizzly bear locations used within our habitat modeling process. We counted all fruits regardless of phenology within the perimeter of the frame. Transect sites were revisited annually from 2010 to 2021, and counts were conducted when ≥ 50% of fruit was ripe. If no portion of a huckleberry plant was intercepted, the frame was advanced at 0.5 m intervals and empty frames were counted. Fifty frames containing the desired species were counted on each transect. We included empty plot frames in all berries‐per‐unit‐area calculations.

For energetic calculations, we assumed 0.4 g fresh weight per huckleberry and 0.494 kcal (kcal) digestible energy per fresh gram (Haytowitz et al. 2019; Pritchard and Robbins 1990; Welch et al. 1997). Daily intake rates were calculated, assuming a minimum 16 active hours per day, 80% of which were spent foraging (Gebhard 1982; Hechtel 1985; Stelmock and Dean 1986; Welch et al. 1997). Simplified energetic requirements (kcal per day) were calculated as the sum of: (1) basal metabolic rate, (2) cost of standing, and (3) cost of moving (1, 3, 6, and 10 km per day) (Kleiber 1932; Robbins 1993). All calculations were made per the model bear's specific body mass. Modeled movement rates were based on observed rates from GPS‐collared female grizzly bears in the two populations during huckleberry season.

## 3 Results

### 3.1 Assessment of Berry Feeding Locations

During 2018–2020, we visited and collected data at 683 grizzly bear seasonal locations of 22 female bears to determine which sites were huckleberry use locations. Similar to Proctor et al. (2023), we found expert opinion aligned strongest with the mean percent huckleberry plant canopy coverage among the five microplots at the site (Figure S3; *p* < 0.001). We then applied a conservative huckleberry canopy coverage cutoff (> 12%) to the site visitation dataset, resulting in 431 huckleberry foraging locations (see Supporting Information).

### 3.2 Model Development and Fit

**FIGURE 1:**
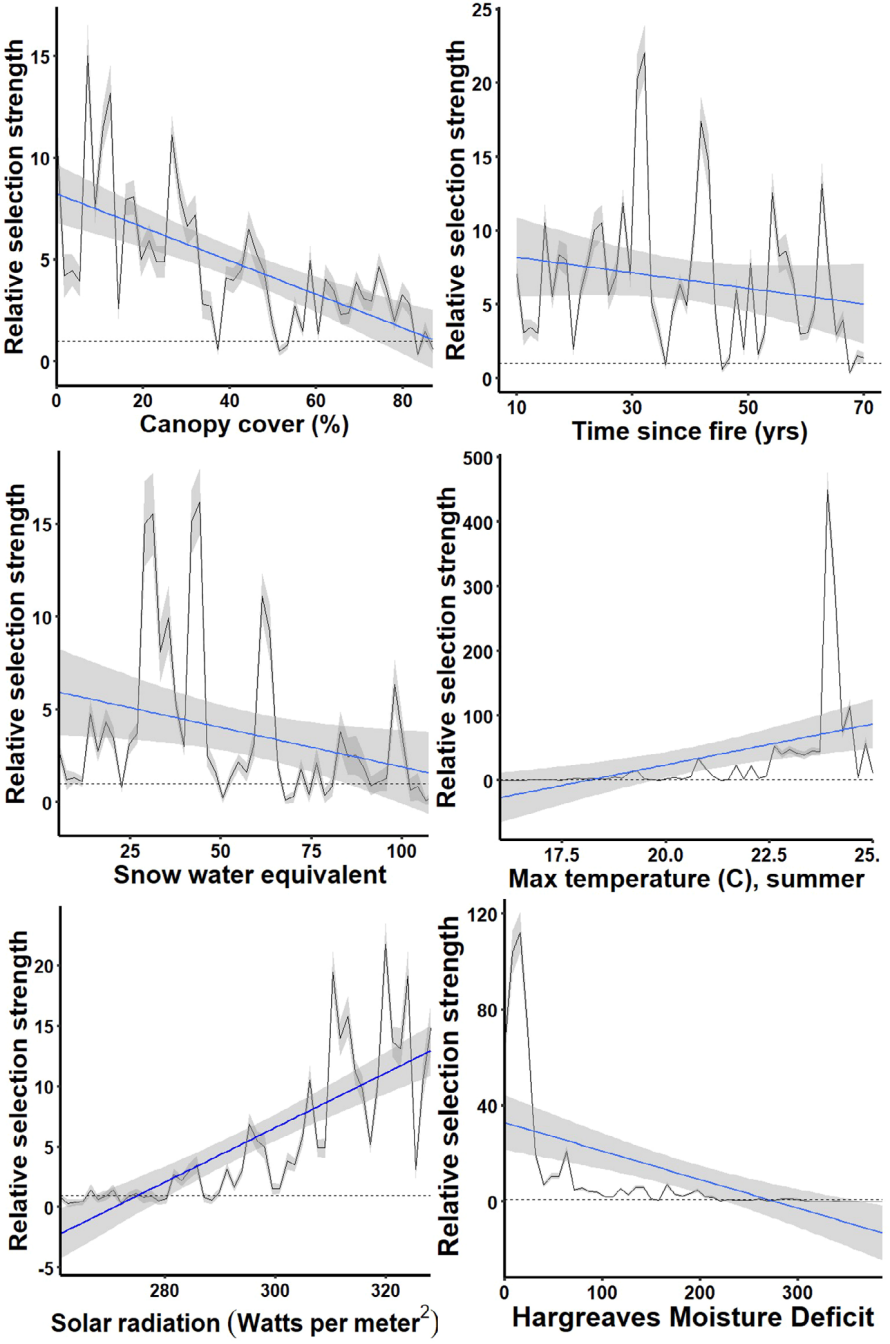
Relative selection strength (RSS) plots across range of values of the six most important variables in predicting huckleberry habitat within the Cabinet‐Yaak and Selkirk Mountains, 2010–2019. CMD is a measure of soil moisture deficit, see Table 1 for more description and details for all variables. Here, RSS scores represent odds of selection, holding all other variables at their average values. The horizontal dotted line depicts neither selection above nor below expected (RSS = 1, or 1:1 odds; Avgar et al. 2017). RSS above 1 indicates selection influence. We provide raw data (in gray) and direct relationship (in blue) for clear interpretation of selection across the range of values. The gray ribbons depict 95% confidence intervals around the raw and smoothed RSS values.

We extracted values for all 28 environmental variables at ground‐truthed huckleberry foraging locations and 4310 available points. Stepwise logistic regression analysis identified 12 variables of statistical significance (*p* < 0.05); all were included in a predictive final model (Table 2; Somers' D = 0.729; K‐S statistic = 0.571, *p* < 0.00001). The Receiver Operating Characteristic (ROC) curve and Area Under the Curve (AUC = 0.865) suggested good to excellent model classification (Figure S4). Top six predictor variables in order of influence included tree canopy cover, time since last fire, Hargreaves Moisture Deficit, mean snow water equivalent, solar radiation, and maximum summer temperature. No variables were significantly correlated (Table 3). Relative selection strength plots had similar relationships as the model coefficients (Figure 1).

**TABLE 2:** Environmental variables included in the best‐fitting model that predicts huckleberry locations used by grizzly bears in the Cabinet‐Yaak and Selkirk study areas, 2010–2019. Coefficients and significance (*p* < 0.05) in the final model are presented.

Variable	Coefficient	Significance	Explanation
Canopy Cover	−0.027	*p* < 0.00001	Tree canopy closure (2016; USFS Landfire)
CMD	−0.020	*p* < 0.00001	Hargreaves Climatic Moisture Deficit (mm)
CTI	−0.118	*p* = 0.011	Compound topographic index
DD5	−0.006	*p* < 0.0001	Degree‐days above 5°C
Last Fire	−0.596	*p* < 0.00001	Time since last fire (years, before 2015)
5‐year Precip	−0.947	*p* < 0.001	Mean annual precipitation (2010–2015)
35‐year Precip	1.113	*p* = 0.012	Mean annual precipitation (1980–2015)
Solar Radiation	0.047	*p* < 0.00001	Mean annual solar radiation (1980–2015)
5‐yr Snowpack	−0.025	*p* < 0.00001	Mean annual snow water equivalent (2010–2015)
Slope	−0.035	*p* < 0.0001	Slope in degrees (°)
Winter Temp	0.560	*p* < 0.001	Average temperature in winter
Summer Temp	0.690	*p* < 0.00001	Maximum temperature in summer

**TABLE 3:** Correlation matrix for all variables included in the final model.

	CMD	cti	DD5	LastFire	Slope	Tave_winter	Tmax_summer	MPrc 10_15	MPrc 80–15	MSRd_80–15	MSWE 10–15	EVC_Tcan
CMD	1.00											
cti	0.05	1.00										
DD5	0.10	−0.01	1.00									
LastFire	0.04	−0.03	0.17	1.00								
Slope	−0.04	0.46	0.02	0.02	1.00							
Tave_winter	0.04	−0.03	−0.50	−0.16	−0.05	1.00						
Tmax_summer	−0.53	−0.06	−0.69	−0.06	0.00	0.01	1.00					
MPrc 10_15	0.07	−0.03	0.33	0.02	−0.11	−0.24	−0.35	1.00				
MPrc 80–15	0.13	0.02	−0.39	−0.05	0.02	0.08	0.36	−0.78	1.00			
MSRd_80–15	−0.41	−0.02	0.18	−0.01	−0.06	−0.10	0.03	0.42	−0.49	1.00		
MSWE 10–15	0.00	−0.03	0.19	0.03	0.05	0.26	−0.04	−0.23	−0.11	−0.26	1.00	
EVC_Tcan	0.01	0.12	0.06	−0.24	0.20	−0.08	−0.10	0.12	−0.04	0.00	−0.01	1.00

We assessed all model fit statistics along the range of log‐odds model values (Table 4). Diagnostic odds ratio at all proposed cutoffs was above 1.0 and indicated positive model performance at all cutoffs. We settled on four cutoff values that represented varying but important levels of model prediction (Table 4). All log‐odds values were transformed to their corresponding explicit likelihood that the cell contains ground‐truthed huckleberry habitat (Figure S2).

**TABLE 4:** Model fit statistics at model values of importance. For each model value, we also present the explicit probability of a grid cell containing a huckleberry feeding site, based on the binned relationship using only our empirical site data collected. Stars in Figure S4 correspond to proposed cutoffs.

Proposed cutoff		Sensitivity, or recall	Specificity	1‐sensitivity, type II error	Sensitivity × specificity	Precision, or user's accuracy	*F*_beta_	Combined accuracy	Diagnostic odds ratio	Importance and rationale
Model log odds value	Empirical probability									
0.030	0.494	0.949	0.487	0.051	0.462	0.156	0.268	0.529	17.6	Type II Error is 0.05. 95% of huck points are correctly predicted. However specificity is low; only 49% of non‐berry points were correctly predicted
0.088	0.590	0.810	0.762	0.190	0.617	0.253	0.390	0.766	13.6	The product of specificity and sensitivity is maximized. Least biased category in terms of true positives and true negatives
0.287	0.711	0.448	0.945	0.552	0.423	0.448	0.448	0.900	13.9	*F*_beta_ maximized and recall/precision are balanced. Least biased category in terms of false positives and false negatives
0.367	0.744	0.355	0.968	0.645	0.343	0.522	0.423	0.912	16.4	Combined accuracy is maximized but false negative rate is high. Prediction is very tight. Locations are highest quality habitat

### 3.3 Predicted Amount of Huckleberry Habitat

We used empirical model probabilities as an estimate of total predicted area of habitat within a cell. Model predicted huckleberry habitat covers 834.7 km^2^ of the US Selkirks study area. The Cabinet‐Yaak study area includes 2203.3 km^2^ of predicted huckleberry habitat (819.5 km^2^ in Yaak; 1383.8 km^2^ in Cabinets). On average, adult female annual home ranges (minimum convex polygon, MCP) include 83.8 ± 8.5 (SE) km^2^ of predicted huckleberry habitat (91.0 ± 7.6 [SE] km^2^ for Cabinet‐Yaak females; 75.5 ± 9.3 [SE] km^2^ for Selkirk females). These numbers translate to approximately 29% of an adult female's home range containing predicted huckleberry habitat (±2.5% [SE]) (Figure S5). Total area and percentage of home range with predicted habitat did not significantly differ between Cabinet‐Yaak and Selkirk adult females (*p* = 0.81; *p* = 0.73).

### 3.4 Degree of Adult Female Overlap Within Predicted Habitat

**FIGURE 2:**
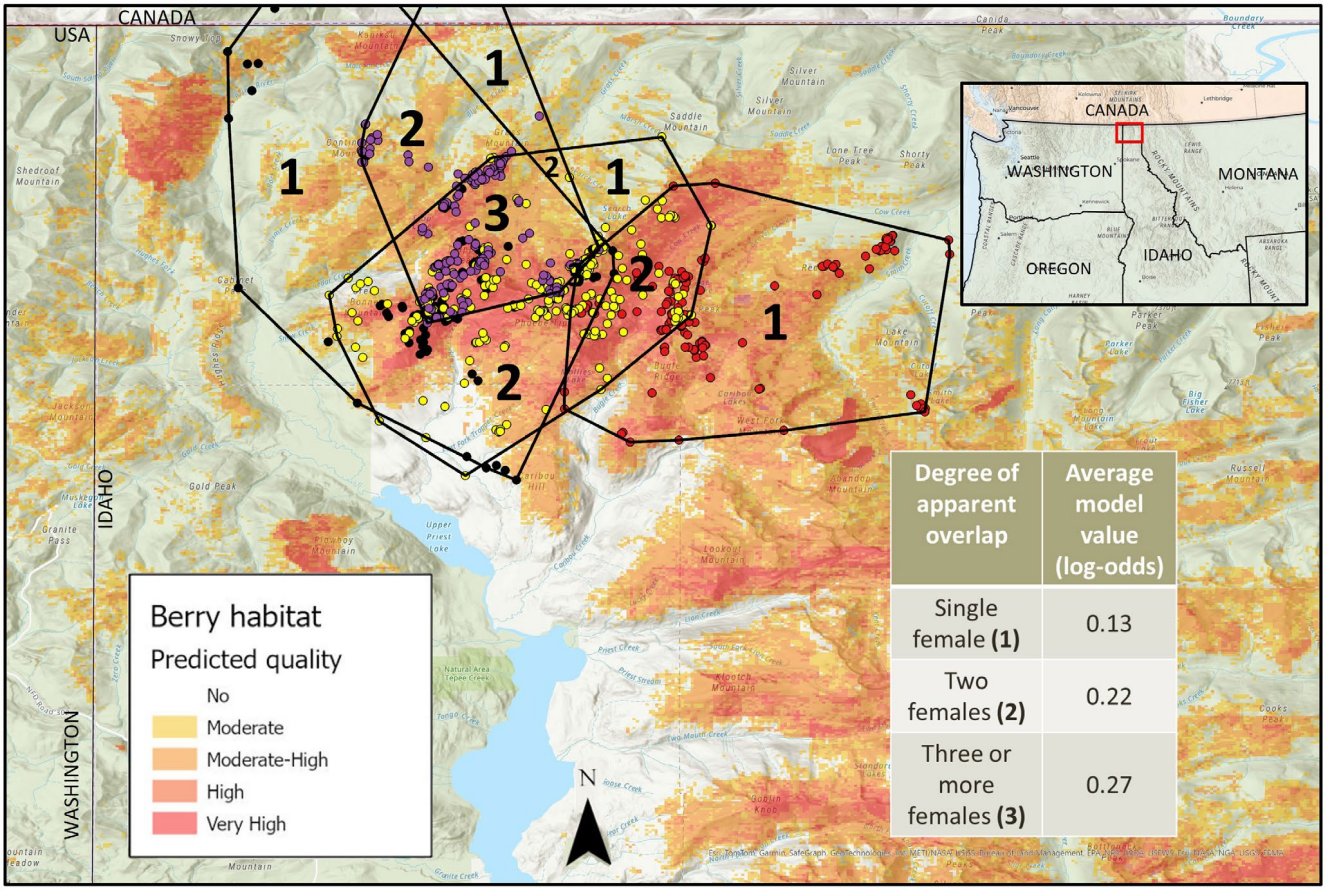
Seasonal overlap within predicted huckleberry habitat of 4 adult female grizzly bears in the Selkirk Mountains, 2013 (#2003 [red], #2008 [black], #2016 [yellow], and #3023 [purple]). Numbers within polygons designate the minimum number of adult female range overlap (via 100% minimum convex polygon). Log‐odds model values for predicted huckleberry habitat summarized by category of overlap. Higher quality habitat is associated with higher degree of female overlap.

Seasonal MCPs of adult females within the same year overlapped widely. While not all female grizzly bears were collared at once, one scenario in particular provided inference on a minimum level of female overlap with respect to predicted berry habitat. Four adult females were known to seasonally overlap in the Selkirks in 2013 (Figure 2). Known seasonal ranges varied from 79.0 to 184.2 km^2^. Of these females, 61.8% (low: 23.2 and high: 89.1%) of one single female seasonal range overlapped with at least one other adult female. Within the berry season of 2013 (July 15—September 15), four collared adult female ranges exhibited a high degree of habitat overlap. Their combined seasonal ranges included 140 km^2^ of predicted berry habitat, with more than half (76 km^2^) of that habitat within all four females' ranges. Further, predicted higher‐quality huckleberry habitat (higher log‐odds model value) experiences a higher degree of female overlap: mean model value of predicted habitat within single female range is 0.13; habitat shared between at least two females has a mean model value of 0.22; habitat shared among three females has a mean model value of 0.27.

### 3.5 Female Habitat Use: Adults vs. Subadults

Both adult females and subadult females ages 3 and 4 (i.e., independent, non‐reproductive females) equally select for predicted huckleberry habitat during berry season (adult female: *mean* model value of seasonal GPS locations 0.203 ± 0.016 [SE], 61 bear‐seasons; subadult female: mean model value 0.210 ± 0.025 [SE], 20 bear‐seasons). Dispersal‐aged, subadult females selected annual home ranges containing 31 ± 2 (SE) percent predicted berry habitat. Mean annual home range of dispersal‐aged females was 33% larger than that of adult females. However, adult female home ranges contained a similar percentage of predicted huckleberry habitat (29 km^2^ ± 3%). Percent predicted habitat between adult mothers and their subadult, independent daughters was not significantly different (mean difference 4.6 ± 2.5 [SE] percent; *N* = 13 dyads; *p* = 0.077).

### 3.6 Berry Production and Predicted Habitat

**FIGURE 3:**
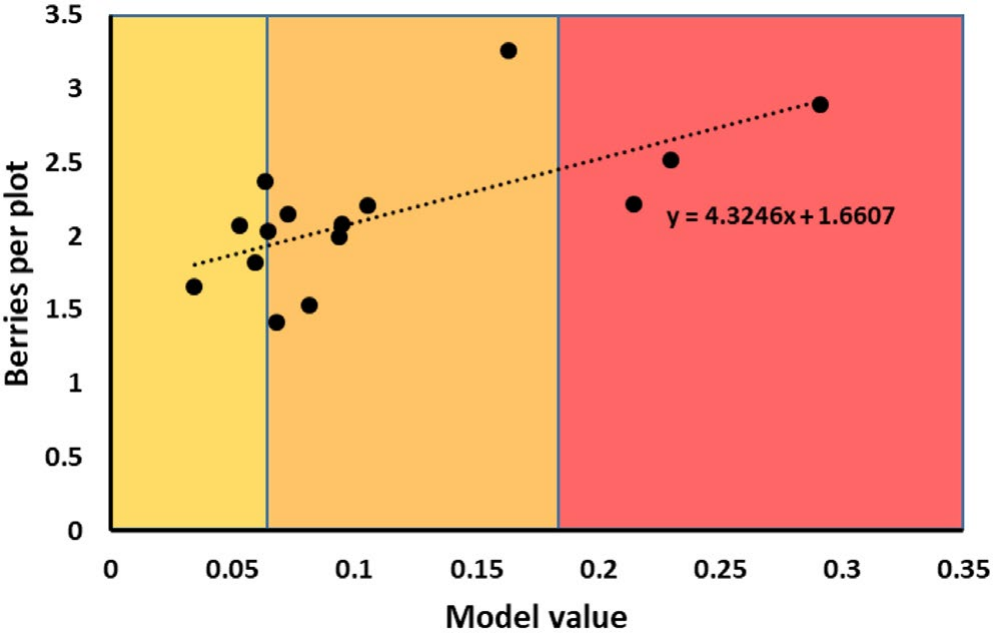
Plot of model value (log‐odds) and average berries per plot (2010–2021), for *N* = 15 independent huckleberry transect sites, monitored for long‐term berry production. All fifteen sites fell within RSF‐predicted berry habitat important to female grizzly bears. Shading indicates model value cutoffs, refer to Table 2. Relationship is significant (General Linear Model; slope or coefficient significantly different from zero, *p* = 0.006).

**FIGURE 4:**
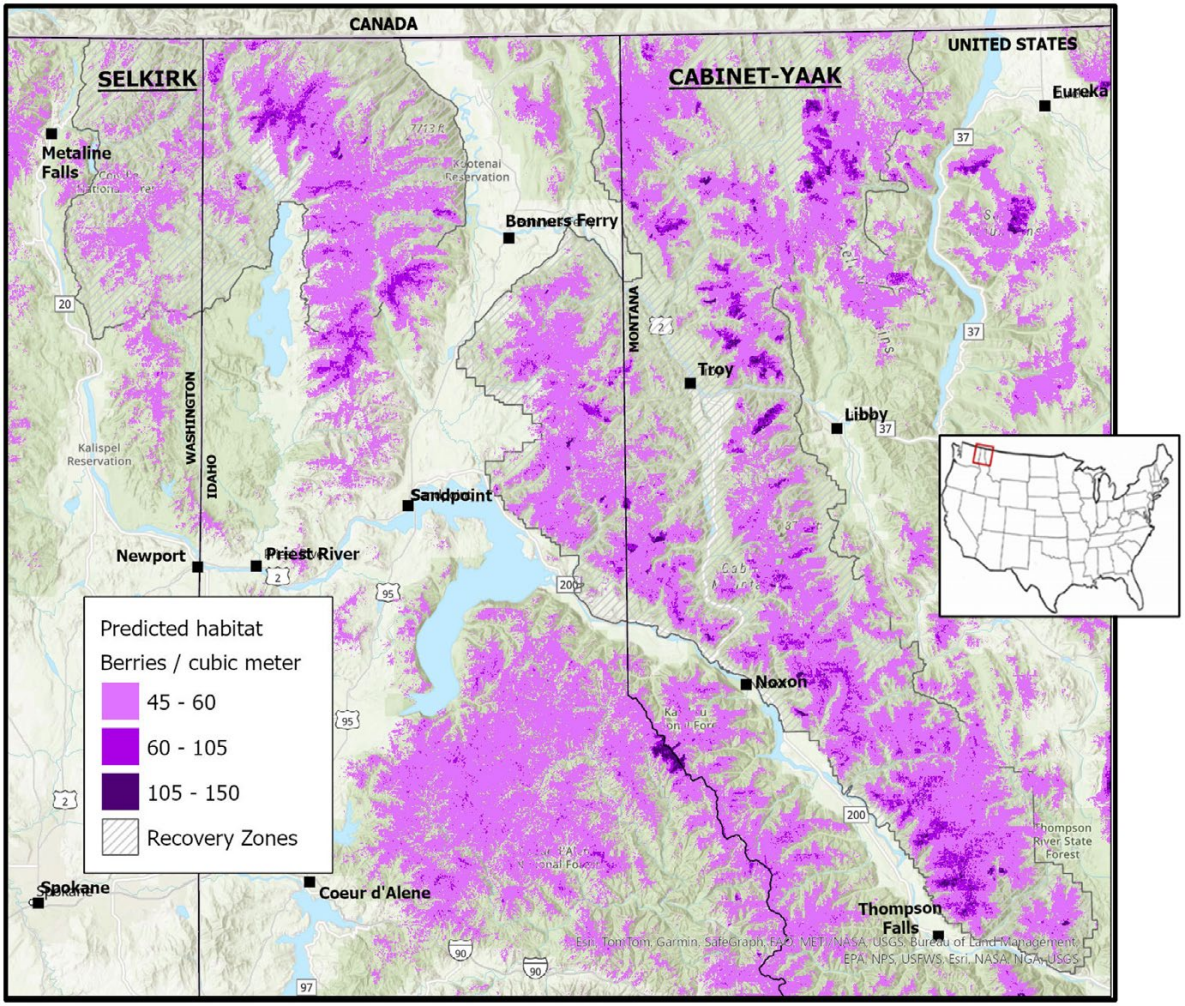
Predicted huckleberry habitat for Cabinet‐Yaak and Selkirk study area. Map predictions include appropriate model value cutoffs (Table 2) and are presented using berry density predictions (Figure 3). Predicted habitat represents timeframe of modeling (2010–2019).

Fifteen separate, independent, long‐term huckleberry transect sites fell within predicted huckleberry habitat and had sufficient data to obtain a mean value of huckleberries per plot, 2010–2021. Eight of the long‐term transect sites fell outside of predicted habitat and were not considered within our productivity modeling. Mean number of berries per plot (0.04 m^2^) for all 15 sites was 2.14 ± 0.13 (SE) berries (Kasworm et al. 2024b). Productivity varied widely among sites, suggesting this wide degree of difference adequately represented that of the landscape. Model log‐odds selection ratio was a significant predictor of berry production (berries per plot) at these 15 sites (Figure 3; Coefficient = 4.325 ± 1.306 [SE]; *p* = 0.006). We applied this significant linear regression function to our predictions of huckleberry habitat area. This produced a map with landscape‐level, long‐term‐average berry abundance within predicted huckleberry habitat (Figure 4). Cell resolution was maintained at 100 m x 100 m. We estimate between 4 and 5.75 billion berries within predicted huckleberry habitat of an average adult female annual home range in the Cabinet‐Yaak and Selkirks (*x¯* = 4.8, SE ± 0.495, *N* = 64, 2006–2021). Mean berry densities within huckleberry habitat ranged from 50.5 to 73.0 berries per m^3^ within female annual MCPs. Our estimates of berries translate into an average 0.956 ± 0.098 (SE) billion kcal available within a female annual range.

## 4 Discussion

### 4.1 Huckleberry Habitat—Findings and Modeling Limitations

We demonstrate that huckleberries, while a seasonal dietary component, are an important landscape driver for these two small populations of grizzly bears. Compared to other grizzly bear populations in the lower‐48 States, Cabinet‐Yaak and Selkirk grizzly bears likely have a greater ecosystem‐ or population‐wide reliance on berry food resources (Aune and Kasworm 1989; Gunther et al. 2014; Mace and Jonkel 1986). Considering habitat within female ranges and holding all factors constant with current conditions (i.e., inter‐specific competition, black bear density, male–female competition, male grizzly bear density, female range size and huckleberry use), we estimate that predicted huckleberry habitat within the U.S. Selkirks could support a minimum of 37 females (subadult and adult). That same set of assumptions suggests a minimum of 82 subadult and adult females could be supported in the Cabinet‐Yaak. Assuming stable age distributions, these numbers translate into roughly twice those of current recovery targets for the two populations and fall within density estimates for biological carrying capacity in this and other ecosystems (Kendall et al. 2019; Turnock 2024). These estimates assume entire segregation (i.e., no overlap) of predicted berry habitat among females. A moderate level of habitat and resource overlap among females, as reported herein, would increase the number of bears supported.

Subadult females (ages 3 and 4) selected for predicted habitat (1) on par with adult females and (2) far greater than what is available within the two ecosystems. In all known mother‐daughter dyads, dispersal‐aged females displayed some degree of overlap with their mother's known range, yet their range outside their mother's had a largely similar composition of huckleberry habitat. While McLellan and Hovey (2001) did not include habitat quality as an influence, they found a similar pattern of mother‐female offspring overlap during multi‐year dispersal. Predicted berry habitat appeared to be an important element of range selection by subadult females and offers seasonal insight into directionality of female range expansion, potential linkage areas, and possible areas of current or future human‐bear conflict (Figure S7). As an important element of dispersal and range expansion, Proctor et al. (2015) demonstrated that linkage areas across highways were best predicted when in proximity to and largely connecting productive, high‐quality habitat (Figure S8). However, improper sanitation in linkage areas can preclude connectivity and create attractive sinks (Lamb et al. 2016; Proctor et al. 2015).

Our modeling process intentionally left out features of human presence (i.e., open roads, developed areas or structures). Instead, we focused on ecological characteristics thought to influence bottom‐up productivity of huckleberries alone. On a population level, grizzly bear avoidance of roads is largely a product of higher survival, fitness, and density of females who select habitat away from roads (Proctor et al. 2020, 2023). Indeed, some seasonal bear locations are near roads, even though female grizzly bears select habitat near roads less than expected (Proctor et al. 2015; Wakkinen and Kasworm 1997). Our habitat predictions include areas near open roads, suggesting possible intra‐population sink dynamics (i.e., an “ecological trap”; Lamb et al. 2016), where productive huckleberry habitat intersects with the top‐down effects of human‐caused mortality near open roads (Figure S9). Nutritionally, we would expect bears to maximize foraging by selecting berry habitat where density of berries is higher. By selecting habitat on the basis of food resource abundance alone, without a cue to avoid the trade‐off of higher chance of mortality, bears may maladaptively select some habitat patches near roads (Lamb et al. 2016). Proctor et al. (2023) modeled huckleberry patches important for grizzly bears in these ecosystems north of the border in British Columbia, Canada. They found that patches of otherwise high‐quality habitat, but within 500 m of an open road, did not make a significant contribution to the density of the local grizzly bear population. All combined, these types of areas could be a focus for future access management efforts (Proctor et al. 2023).

Similar to our findings, Proctor et al. (2023) found that tree canopy cover is a significant predictor of huckleberry habitat for grizzly bears, in their case < 50% canopy coverage. Grizzly bears in our study area showed moderate selection for canopy coverage out to approximately 70% (Figure 1), and this difference may be due to characteristic differences in huckleberry habitat between the two study areas. The Selkirks of British Columbia include more high‐elevation, large, contiguous shrubfields relative to the smaller habitat patch size in other lower elevation study areas, such as the Yaak. Wildfire and forestry management practices (prescribed fire, intensive “regeneration” timber harvest, and intermediate shelterwood harvest) can open forest canopy, potentially increasing berry habitat available to bears (Souliere et al. 2020). While opening canopy could benefit bottom‐up food production, the potential costs of increasing top‐down mortality with any actions need to be considered.

**FIGURE 5:**
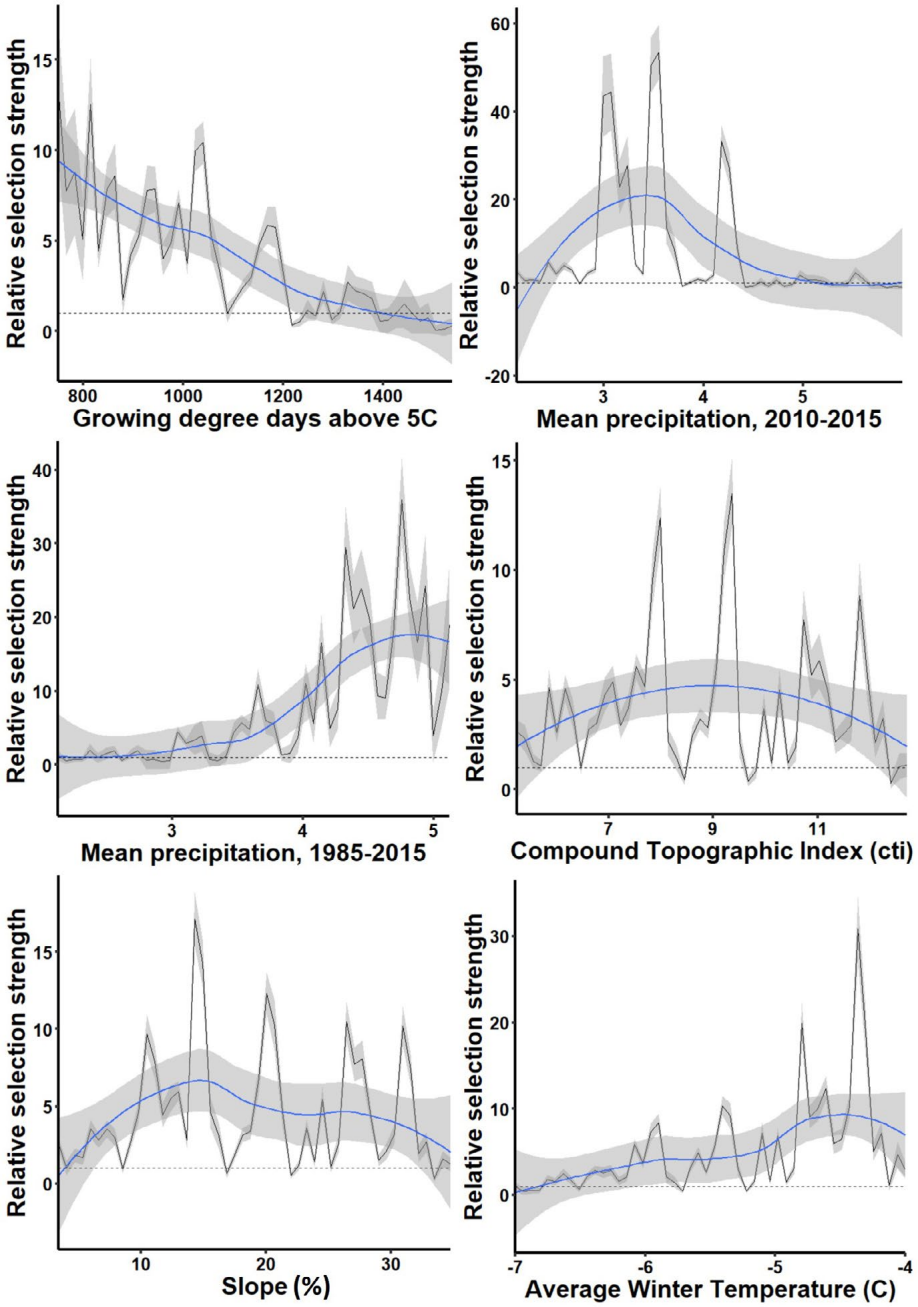
Relative selection strength (RSS) plots across range of values of variables 7–12 in the selected huckleberry habitat model, Cabinet‐Yaak and Selkirk Mountains, 2010–2019. Precipitation is measured as mean annual inches of rain and snow water equivalent, over 5‐ and 30‐year period. See Table 1 for more description and details for variables. Here, RSS scores represent odds of selection, holding all other variables at their average values. The horizontal dotted line depicts neither selection above nor below expected (RSS = 1, or 1:1 odds; Avgar et al. 2017). RSS above one indicates selection influence. We provide a smoothed line (in blue) for easier interpretation of selection across the range of values. The gray ribbons depict 95% confidence intervals around the raw and smoothed RSS values.

Future climatic patterns could shift availability of this important food resource (Prevéy et al. 2020). Locally, warmer spring temperatures result in decreases and greater stochasticity in annual berry productivity (Holden et al. 2012). Alternatively, future climate scenarios may increase the amount of berry habitat. Holden et al. (2012) also found that high mid‐ to late‐summer diurnal temperature ranges are associated with higher huckleberry productivity. We find the same with our modeling; higher maximum summer temperatures are associated with high‐quality huckleberry habitat for grizzly bears (Figure 1). Ransom et al. (2023) found future climate scenarios will likely increase the amount of high elevation meadow and *Vaccinium* spp. habitat for North Cascades grizzly bears. Future climate modeling in the interior northwest predicts warmer temperatures and higher precipitation overall, but less of it in the form of snow (Rupp et al. 2017). Our model outputs would predict more huckleberry habitat under scenarios with warmer winter temperatures, less snowpack (i.e., lower snow water equivalent), and higher summer maximum temperatures (Figures 1 and 5).

Our primary intent was to produce a model with high predictive power. Given this, we prioritized prediction, allowing a higher threshold for correlation coefficients between variables (> 0.8 or < 0.8). We therefore caution over‐interpretation of moderately correlated explanatory variables, such as the negative trend in short‐term and long‐term precipitation. We also did not model interannual variation in berry productivity, though studies have suggested links between low natural food availability and increased human‐bear conflict (Gunther et al. 2004). Future modeling and analyses could focus on nutritional implications of below‐ or above‐average berry years. Similarly, in years of lower huckleberry production, bears may turn to other natural foods with less foraging constraints, such as serviceberry, chokecherry, fungi, invertebrates, or fungi (Fortin et al. 2013; Kasworm et al. 2024a). We also did not include spatial measures or indices of green vegetation, for which the model could have been improved. However, seasonal values for Normalized Difference Vegetation Index (NDVI), a common remote sensing measure of vegetation density (Rouse et al. 1974), associated poorly with predicted huckleberry habitat (*R* ^2^ = 0.028; Figure S10). Greenness is a remotely sensed measure of fresh, vegetative biomass that does not include late‐season, evergreen conifers (Crist and Cicone 1984). Seasonal values of greenness also had very little association with huckleberry model values (*R* ^2^ = 0.055; Figure S11). Future modeling predictions could include (1) intra‐annual tracking of growing degree days and (2) more refined hyperspectral data, with both additions aligning with specific phenological stages for huckleberry (Shores et al. 2019).

### 4.2 Energetics of Berry Foraging—Body Size Implications

We modeled caloric content of berry habitat by aligning our habitat models with multi‐year huckleberry production data at sampling transects. As such, our energetic estimates do not account for interannual variation, inherently representing more of a lifetime average for a bear across the landscape. These predictions may better represent selected habitat by the fittest females and their matrilines (i.e., ≥ 10‐year productivity within predicted habitat is above‐average and most important to females). With our energetic modeling, we recognize that reproductive status of females with young incurs an increased energetic cost beyond our estimates due to gestation and lactation. These higher costs decrease the maximum mass estimates that huckleberry habitat could support. We also recognize that *Vaccinium* spp. are shared as a resource under interspecific (i.e., black bears) and intraspecific scramble competition, and future changes in black bear or grizzly bear densities could negatively or positively influence the amount of available fruit (Mattson et al. 2005). Follow‐up studies are needed. With high densities of black bears, our study area could be one of a few model study systems.

Females appear to be selecting for higher‐quality huckleberry habitat. All huckleberry density values within female ranges were above mean densities measured at berry productivity transects within the Cabinet‐Yaak‐Selkirk (45.0 berries per meter^3^ 1989–2021; Kasworm et al. 2024b). Welch et al. (1997) found that bears were able to “high‐grade” and maximize bite rate when berries were > 50.0 berries per meter^3^. Berry densities modeled within female ranges translate into a realized intake of 6.8 to 8.0 g of fresh weight huckleberries per minute (Welch et al. 1997). Given berry densities reported here, expected mean bite rates in Cabinet‐Yaak and Selkirk ecosystems are between 20 and 23 per minute, similar to those observed with wild grizzly bears in Glacier National Park (Welch et al. 1997). At the upper end of predicted berry densities, bite rates would reach (1) a maximum of 39 bites per minute (asymptotic maximum in Welch et al. 1997) and (2) 11 g wet weight fruit consumed per minute (approximately 5.4 kcal per minute).

**FIGURE 6:**
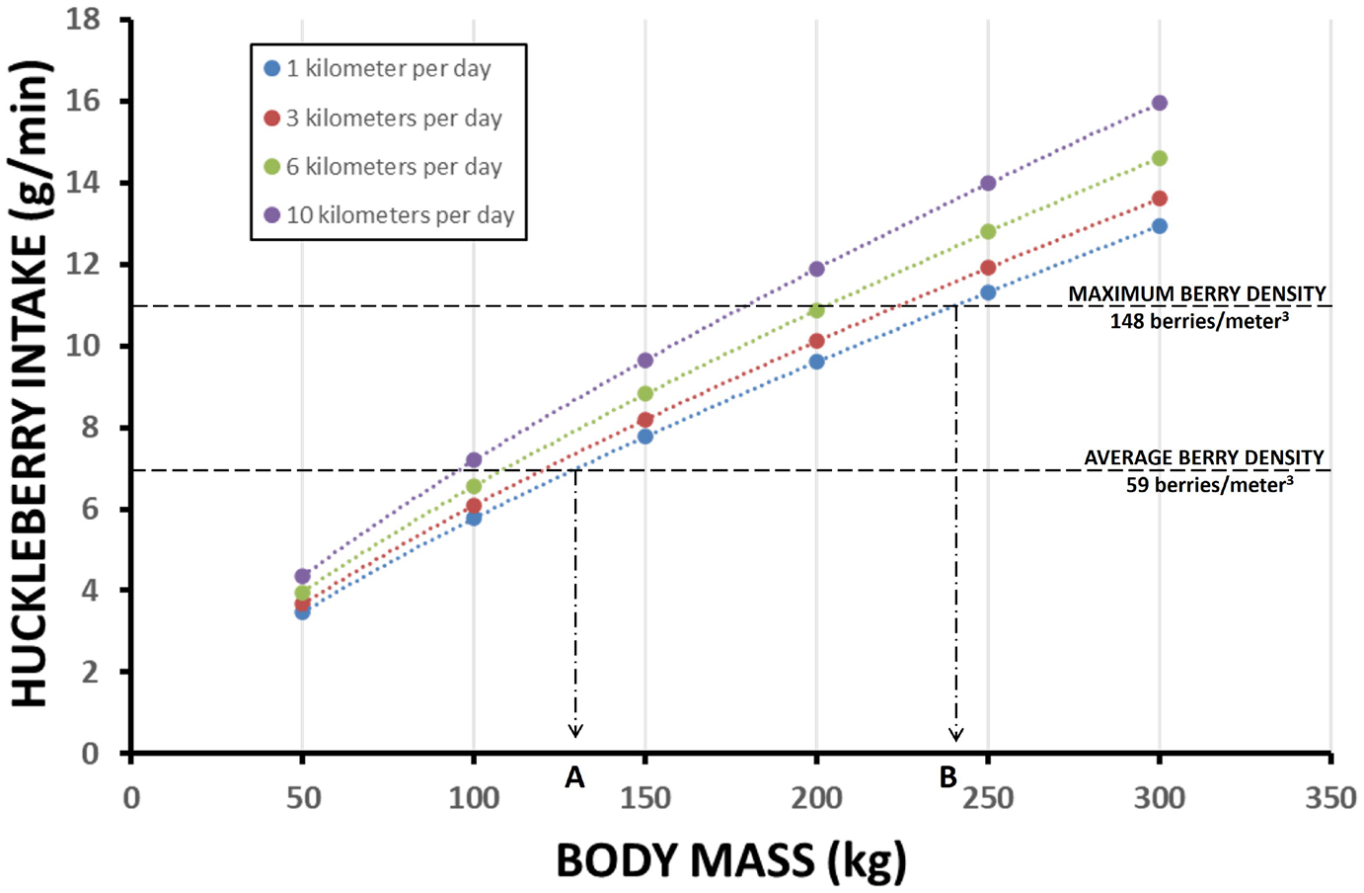
Huckleberry intake (grams per minute [fresh weight]) required for maintenance of bears with varying movement rates (1, 3, 6, and 10 km per day), as a function of body mass (kilograms). Calculations assume 80% of active daily hours (16) dedicated to foraging, and huckleberries are the sole source of energy (see text). Expected huckleberry intake rates are plotted. Theoretical intake rates were calculated using predicted average and maximum density of berries from model outputs applied to curvilinear relationships developed with captive bear feeding trials (Welch et al. 1997). A bear weighing < 130 kg foraging within average huckleberry density in the Cabinet‐Yaak and Selkirk is generally expected to meet energy requirements on huckleberries alone. (A) A bear > 240 kg cannot be energetically supported by huckleberries alone. (B) For bears between 130 kg and 240 kg, mass gain or loss would depend largely on movement rate and berry density.

Calculations of energy intake were assessed relative to body mass and expected energy expenditures (Figure 6). Bears < 125 kg are expected to gain mass at all predicted berry densities within Cabinet‐Yaak‐Selkirk habitat. Only 14% of adult females caught in the Selkirk‐Cabinet‐Yaak weighed above 125 kg, and nearly all of those were fall measures of fat bears, where lean body mass would be far less than 125 kg. Bears weighing greater than 240 kg can break even calorically on a diet predominantly composed of huckleberries if they maximize energetic efficiency (Figure 6). However, only 4 grizzly bears captured in the Selkirk‐Cabinet‐Yaak approached or exceeded 240 kg. Interestingly, all 4 were previously involved in human‐bear conflicts with highly‐attractive, human‐associated foods (beehives, small livestock, garbage). These findings suggest independent females < 125 kg will gain body mass during berry season, when huckleberries are a main source of calories. For bears between 125 and 240 kg, mass gain or loss would depend on movement rates, habitat selection, and annual local huckleberry productivity.

Selection for body sizes with less energetic demand could occur in Cabinet‐Yaak and Selkirk populations, given huckleberries are a primary food source in late summer and early fall. Smaller‐sized bears are better suited in food economies with lesser nutritional quality, or those with foods that have foraging constraints (Costello et al. 2015; Matsubayashi et al. 2016; Welch et al. 1997). When food resources are limited in either quality or quantity, smaller size can confer a fitness advantage by reducing energy requirements while allowing for higher levels of body fat accumulation that would not be possible in larger bears (Cameron et al. 2020; McLellan 2011). Nutrition acquired in subadult years of life (typically, birth to 5 years of age) strongly influences structural body size as an adult, and energetic demand is positively related to body size. Hence, a female's productivity may rely on whether foods available in adulthood match those acquired when the bear was dependent upon its mother or dispersing to establish their own adult range. One can also theorize potential, negative “mismatches”, situations where resources available under‐nourish the bear. As such, we hypothesize structural body size, female density, and female fitness are, in part, dependent upon food density and overall amount within her range. McLellan (2011) found that a huckleberry‐dominant system in British Columbia to the northeast of our study area (NF Flathead drainage) produced “smaller” bears relative to coastal salmon or heavy meat‐dominated systems, but still at relatively high densities (McLellan 2015). At current densities, Cabinet‐Yaak and Selkirk grizzly bear reproductive rates are similar to those of other populations in the lower‐48 United States (Costello and Roberts 2019; Kasworm et al. 2024a, 2024b; Schwartz and White 2008). Altogether, the generalist, omnivorous foraging strategy and smaller body size of Cabinet‐Yaak and Selkirk grizzly bears may benefit these populations under future, unknown food economies.

## Author Contributions

**Justin E. Teisberg:** conceptualization (equal), data curation (equal), formal analysis (equal), investigation (equal), methodology (equal), resources (equal), software (equal), validation (equal), visualization (equal), writing – original draft (equal), writing – review and editing (equal). **Wayne F. Kasworm:** conceptualization (equal), data curation (equal), funding acquisition (equal), investigation (equal), methodology (equal), project administration (equal), resources (equal), supervision (equal), writing – original draft (equal), writing – review and editing (equal). **Michael F. Proctor:** conceptualization (equal), formal analysis (equal), methodology (equal), resources (equal), software (equal), validation (equal), writing – review and editing (equal). **Thomas G. Radandt:** conceptualization (equal), data curation (equal), formal analysis (equal), investigation (equal), methodology (equal), resources (equal), validation (equal), writing – review and editing (equal). **Jennifer K. Fortin‐Noreus:** conceptualization (equal), data curation (equal), methodology (equal), resources (equal), writing – review and editing (equal). **Hilary S. Cooley:** conceptualization (equal), funding acquisition (equal), project administration (equal), resources (equal), supervision (equal), writing – review and editing (equal).

## Funding

This work was supported by the US Fish and Wildlife Service and University of Montana.

## Conflicts of Interest

The authors declare no conflicts of interest.

## Supporting information


**Data S1:** ece372905‐sup‐0001‐Supinfo.docx.

## Data Availability

The data underlying the findings of this study are available within Dryad, an open data repository https://doi.org/10.5061/dryad.w6m905r33. Locations have been anonymized and rounded to the nearest 100 km. Under United States statute, limitations apply with providing precise GPS or VHF telemetry locations from individual animals of this sensitive, federally listed species.

## References

[ece372905-bib-0001] Aune, K. , and W. Kasworm . 1989. Final Report: East Front Grizzly Bear Studies. Montana Department of Fish, Wildlife and Parks.

[ece372905-bib-0002] Avgar, T. , S. R. Lele , J. L. Keim , and M. S. Boyce . 2017. “Relative Selection Strength: Quantifying Effect Size in Habitat‐ and Step‐Selection Inference.” Ecology and Evolution 7, no. 14: 5322–5330. 10.1002/ece3.3122. 28770070 PMC5528224

[ece372905-bib-0003] Barney, D. 1999. Growing Western Huckleberries. University of Idaho, Sandpoint Research Extension Center.

[ece372905-bib-0004] Barney, D. , P. McDaniel , and A. Falen . 2006. “Soil Characteristics Associated With Wild Huckleberry and Bilberry Colonies in the Northwestern United States: Implications for Managed Production and Cultivation.” Proceedings of the North American Blueberry Research & Extension Workers Conference.

[ece372905-bib-0005] Boyce, M. S. 2006. “Scale for Resource Selection Functions.” Diversity and Distributions 12, no. 3: 269–276.

[ece372905-bib-0006] Boyce, M. S. , and L. L. McDonald . 1999. “Relating Populations to Habitats Using Resource Selection Functions.” Trends in Ecology & Evolution 14, no. 7: 268–272. 10370262 10.1016/s0169-5347(99)01593-1

[ece372905-bib-0007] Boyce, M. S. , P. R. Vernier , S. E. Nielsen , and F. K. A. Schmiegelow . 2002. “Evaluating Resource Selection Functions.” Ecological Modelling 157, no. 2–3: 281–300.

[ece372905-bib-0008] Cameron, M. D. , G. V. Hilderbrand , K. Joly , et al. 2020. “Body Size Plasticity in North American Black and Brown Bears.” Ecosphere 11, no. 8: e03235. 10.1002/ecs2.3235.

[ece372905-bib-0009] Ciarniello, L. M. , M. S. Boyce , D. R. Seip , and D. C. Heard . 2007. “Grizzly Bear Habitat Selection Is Scale Dependent.” Ecological Applications 17, no. 5: 1424–1440. 17708219 10.1890/06-1100.1

[ece372905-bib-0010] Costello, C. M. , S. L. Cain , S. Pils , L. Frattaroli , M. A. Haroldson , and F. T. van Manen . 2015. “Diet and Macronutrient Optimization in Wild Ursids: A Comparison of Grizzly Bears With Sympatric and Allopatric Black Bears.” PLoS One 11, no. 5: e0153702. 10.1371/journal.pone.0153702. PMC4871523 27192407

[ece372905-bib-0011] Costello, C. M. , and L. L. Roberts . 2019. “Northern Continental Divide Ecosystem Grizzly Bear Population Monitoring Team Annual Report–2019.”

[ece372905-bib-0012] Crist, E. P. , and R. C. Cicone . 1984. “A Physically‐Based Transformation of Thematic Mapper Data—The TM Tasseled Cap.” IEEE Transactions on Geoscience and Remote Sensing GE‐22, no. 3: 256–263. 10.1109/TGRS.1984.350619.

[ece372905-bib-0013] Dahle, B. , and J. E. Swenson . 2003. “Home Ranges in Adult Scandinavian Brown Bears (*Ursus arctos*): Effect of Mass, Sex, Reproductive Category, Population Density and Habitat Type.” Journal of Zoology 260, no. 4: 329–335.

[ece372905-bib-0014] Denny, C. K. , G. B. Stenhouse , and S. E. Nielsen . 2018. “Scales of Selection and Perception: Landscape Heterogeneity of an Important Food Resource Influences Habitat Use by a Large Omnivore.” Wildlife Biology 2018, no. 1: 1–10. 10.2981/wlb.00409.

[ece372905-bib-0015] Edwards, M. A. , A. E. Derocher , and J. A. Nagy . 2013. “Home Range Size Variation in Female Arctic Grizzly Bears Relative to Reproductive Status and Resource Availability.” PLoS One 8, no. 7: e68130. 10.1371/journal.pone.0068130. 23844162 PMC3700869

[ece372905-bib-0016] Erlenbach, J. A. , K. D. Rode , D. Raubenheimer , and C. T. Robbins . 2014. “Macronutrient Optimization and Energy Maximization Determine Diets of Brown Bears.” Journal of Mammalogy 95, no. 1: 160–168. 10.1644/13-MAMM-A-161.

[ece372905-bib-0017] Felicetti, L. A. , C. T. Robbins , and L. A. Shipley . 2003. “Dietary Protein Content Alters Energy Expenditure and Composition of the Mass Gain in Grizzly Bears ( *Ursus arctos horribilis* ).” Physiological and Biochemical Zoology 76, no. 2: 256–261. 12794679 10.1086/374279

[ece372905-bib-0018] Fortin, J. K. , C. C. Schwartz , K. A. Gunther , et al. 2013. “Dietary Adjustability of Grizzly Bears and American Black Bears in Yellowstone National Park.” Journal of Wildlife Management 77, no. 2: 270–281. 10.1002/jwmg.483.

[ece372905-bib-0019] Gebhard, J. G. 1982. Annual Activities and Behavior of a Grizzly Bear (Ursus arctos) Family in Northern Alaska. University of Alaska Fairbanks.

[ece372905-bib-0020] Guillera‐Arroita, G. , J. J. Lahoz‐Monfort , J. Elith , et al. 2015. “Is My Species Distribution Model Fit for Purpose? Matching Data and Models to Applications.” Global Ecology and Biogeography 24, no. 3: 276–292.

[ece372905-bib-0021] Gunther, K. A. , S. L. Cain , J. Copeland , K. Frey , M. A. Haroldson , and C. C. Schwartz . 2004. “Grizzly Bear‐Human Conflicts in the Greater Yellowstone Ecosystem, 1992‐2000.” Ursus 15, no. 1: 10–22.

[ece372905-bib-0022] Gunther, K. A. , R. R. Shoemaker , K. L. Frey , et al. 2014. “Dietary Breadth of Grizzly Bears in the Greater Yellowstone Ecosystem.” Ursus 25, no. 1: 60. 10.2192/URSUS-D-13-00008.1.

[ece372905-bib-0023] Hanley, J. A. , and B. J. McNeil . 1982. “The Meaning and Use of the Area Under a Receiver Operating Characteristic (ROC) Curve.” Radiology 143, no. 1: 29–36. 7063747 10.1148/radiology.143.1.7063747

[ece372905-bib-0024] Haytowitz, D. B. , J. K. Ahuja , X. Wu , et al. 2019. USDA National Nutrient Database for Standard Reference, Legacy Release. Nutrient Data Laboratory, Beltsville Human Nutrition Research Center, ARS, USDA.

[ece372905-bib-0025] Hechtel, J. L. 1985. Activity and Food Habits of Barren‐Ground Grizzly Bears in Arctic Alaska. University of Montana.

[ece372905-bib-0026] Hellgren, E. C. , D. W. Carney , N. P. Garner , and M. R. Vaughan . 1988. “Use of Breakaway Cotton Spacers on Radio Collars.” Wildlife Society Bulletin 16, no. 2: 216–218.

[ece372905-bib-0027] Hertel, A. G. , R. Bischof , O. Langval , et al. 2018. “Berry Production Drives Bottom‐Up Effects on Body Mass and Reproductive Success in an Omnivore.” Oikos 127, no. 2: 197–207. 10.1111/oik.04515.

[ece372905-bib-0028] Hilderbrand, G. V. , D. D. Gustine , K. Joly , et al. 2019. “Influence of Maternal Body Size, Condition, and Age on Recruitment of Four Brown Bear Populations.” Ursus 29, no. 2: 111–118. 10.2192/URSUS-D-18-00008.1.

[ece372905-bib-0029] Hilderbrand, G. V. , D. D. Gustine , B. A. Mangipane , et al. 2017. “Body Size and Lean Mass of Brown Bears Across and Within Four Diverse Ecosystems.” Journal of Zoology 10: 1–10. 10.1111/jzo.12536.

[ece372905-bib-0030] Hilderbrand, G. V. , C. C. Schwartz , C. T. Robbins , et al. 1999. “The Importance of Meat, Particularly Salmon, to Body Size, Population Productivity, and Conservation of North American Brown Bears.” Canadian Journal of Zoology 77, no. 1: 132–138.

[ece372905-bib-0031] Hobby, T. , and M. E. Keefer . 2010. “A Black Huckleberry Case Study in the Kootenay Region of British Columbia.” BC Journal of Ecosystems and Management 11: 11.

[ece372905-bib-0032] Holden, Z. A. , W. F. Kasworm , C. Servheen , B. Hahn , and S. Dobrowski . 2012. “Sensitivity of Berry Productivity to Climatic Variation in the Cabinet–Yaak Grizzly Bear Recovery Zone, Northwest United States, 1989–2010.” Wildlife Society Bulletin 36: 226–231. 10.1002/wsb.128.

[ece372905-bib-0033] Johnson, D. H. 1980. “The Comparison of Usage and Availability Measurements for Evaluating Resource Preference.” Ecology 61, no. 1: 65–71.

[ece372905-bib-0089] Johnson, K. G. , and M. R. Pelton . 1980. “Prebaiting and Snaring Techniques for Black Bears.” Wildlife Society Bulletin 8, no. 1: 46–54.

[ece372905-bib-0090] Jonkel, J. 1993. “A Manual for Handling Bears for Managers and Researchers.” Office of Grizzly Bear Recovery Coordinator, U.S. Fish and Wildlife Service, University of Montana.

[ece372905-bib-0034] Kasworm, W. F. , T. G. Radandt , J. E. Teisberg , et al. 2024a. Cabinet‐Yaak Grizzly Bear Recovery Area 2023 Research and Monitoring Progress Report (p. 121). U.S. Fish and Wildlife Service.

[ece372905-bib-0035] Kasworm, W. F. , T. G. Radandt , J. E. Teisberg , et al. 2024b. Selkirk Mountains Grizzly Bear Recovery Area 2023 Research and Monitoring Progress Report (p. 88). U.S. Fish and Wildlife Service.

[ece372905-bib-0036] Kendall, K. C. 1986. Grizzly and Black Bear Feeding Ecology in Glacier National Park, Montana: Progress Report. Science Center, Glacier National Park Biosphere Reserve.

[ece372905-bib-0037] Kendall, K. C. , T. A. Graves , J. A. Royle , et al. 2019. “Using Bear Rub Data and Spatial Capture‐Recapture Models to Estimate Trend in a Brown Bear Population.” Scientific Reports 9: 12. 10.1038/s41598-019-52783-5. 31727927 PMC6856102

[ece372905-bib-0038] Kingsley, M. C. S. , J. A. Nagy , and H. V. Reynolds . 1988. “Growth in Length and Weight of Northern Brown Bears: Differences Between Sexes and Populations.” Canadian Journal of Zoology 66, no. 4: 981–986.

[ece372905-bib-0039] Kleiber, M. 1932. “Body Size and Metabolism.”

[ece372905-bib-0040] Lamb, C. T. , G. Mowat , B. N. McLellan , S. E. Nielsen , and S. Boutin . 2016. “Forbidden Fruit: Human Settlement and Abundant Fruit Create an Ecological Trap for an Apex Omnivore.” Journal of Animal Ecology 86, no. 1: 55–65. 10.1111/1365-2656.12589. 27677529

[ece372905-bib-0041] Leroux, S. J. 2019. “On the Prevalence of Uninformative Parameters in Statistical Models Applying Model Selection in Applied Ecology.” PLoS One 14, no. 2: e0206711. 10.1371/journal.pone.0206711. 30730890 PMC6366740

[ece372905-bib-0042] Lyons, A. L. , W. L. Gaines , P. H. Singleton , W. F. Kasworm , M. F. Proctor , and J. Begley . 2018. “Spatially Explicit Carrying Capacity Estimates to Inform Species Specific Recovery Objectives: Grizzly Bear (*Ursus arctos*) Recovery in the North Cascades.” Biological Conservation 222: 21–32. 10.1016/j.biocon.2018.03.027.

[ece372905-bib-0043] Mace, R. D. , and C. J. Jonkel . 1986. “Local Food Habits of the Grizzly Bear in Montana.” International Conference on Bear Research and Management 6: 105–110.

[ece372905-bib-0044] Mangipane, L. S. , D. J. Lafferty , K. Joly , et al. 2020. “Dietary Plasticity and the Importance of Salmon to Brown Bear (*Ursus arctos*) Body Size and Condition in a Low Arctic Ecosystem.” Polar Biology 43. 10.1007/s00300-020-02690-7.

[ece372905-bib-0045] Manly, B. , L. McDonald , D. L. Thomas , T. L. McDonald , and W. P. Erickson . 2007. Resource Selection by Animals: Statistical Design and Analysis for Field Studies. Springer Science & Business Media.

[ece372905-bib-0046] Matsubayashi, J. , I. Tayasu , J. O. Morimoto , and T. Mano . 2016. “Testing for a Predicted Decrease in Body Size in Brown Bears (*Ursus arctos*) Based on a Historical Shift in Diet.” Canadian Journal of Zoology 94, no. 7: 489–495. 10.1139/cjz-2016-0046.

[ece372905-bib-0047] Mattson, D. J. , S. Herrero , and T. Merrill . 2005. “Are Black Bears a Factor in the Restoration of North American Grizzly Bear Populations?” Ursus 16, no. 1: 11–30.

[ece372905-bib-0048] McClelland, C. J. R. , C. K. Denny , T. A. Larsen , G. B. Stenhouse , and S. E. Nielsen . 2021. “Landscape Estimates of Carrying Capacity for Grizzly Bears Using Nutritional Energy Supply for Management and Conservation Planning.” Journal for Nature Conservation 62: 126018. 10.1016/j.jnc.2021.126018.

[ece372905-bib-0049] McDonough, T. J. , and A. M. Christ . 2012. “Geographic Variation in Size, Growth, and Sexual Dimorphism of Alaska Brown Bears, *Ursus arctos* .” Journal of Mammalogy 93, no. 3: 686–697. 10.1644/11-MAMM-A-010.1.

[ece372905-bib-0050] McLellan, B. N. 2011. “Implications of a High‐Energy and Low‐Protein Diet on Body Composition, Fitness, and Competitive Abilities of Black (*Ursus americanus*) and Grizzly (*Ursus arctos*) Bears.” Canadian Journal of Zoology 89: 546–558. 10.1139/Z11-026.

[ece372905-bib-0051] McLellan, B. N. 2015. “Some Mechanisms Underlying Variation in Vital Rates of Grizzly Bears on a Multiple Use Landscape.” Journal of Wildlife Management 79, no. 5: 749–765. 10.1002/jwmg.896.

[ece372905-bib-0052] McLellan, B. N. , and F. W. Hovey . 1995. “The Diet of Grizzly Bears in the Flathead River Drainage of Southeastern British Columbia.” Canadian Journal of Zoology 73, no. 4: 704–712. 10.1139/z95-082.

[ece372905-bib-0053] McLellan, B. N. , and F. W. Hovey . 2001. “Natal Dispersal of Grizzly Bears.” Canadian Journal of Zoology 79, no. 5: 838–844.

[ece372905-bib-0054] Minore, D. 1984. “ *Vaccinium membranaceum* Berry Production Seven Years After Treatment to Reduce Overstory Tree Canopies.” Northwest Science 58, no. 3: 208–212.

[ece372905-bib-0055] Nielsen, S. , and C. Nielsen . 2010. A Landscape Analysis of Huckleberry in Southeast British Columbia, Spatial–Temporal Patterns of Forest Fires, and Protocols for Long‐Term Monitoring of Inter‐Annual Variation in Fruit Production for Huckleberry and Buffaloberry. Final Report for Habitat Conservation Trust Foundation, University of Alberta.

[ece372905-bib-0056] Nielsen, S. E. , T. A. Larsen , G. B. Stenhouse , and S. C. P. Coogan . 2016. “Complementary Food Resources of Carnivory and Frugivory Affect Local Abundance of an Omnivorous Carnivore.” Oikos 126, no. 3: 369–380. 10.1111/oik.03144.

[ece372905-bib-0057] Prevéy, J. S. , L. E. Parker , C. A. Harrington , C. T. Lamb , and M. F. Proctor . 2020. “Climate Change Shifts in Habitat Suitability and Phenology of Huckleberry (*Vaccinium membranaceum*).” Agricultural and Forest Meteorology 280: 107803. 10.1016/j.agrformet.2019.107803.

[ece372905-bib-0058] Pritchard, G. T. , and C. T. Robbins . 1990. “Digestive and Metabolic Efficiencies of Grizzly and Black Bears.” Canadian Journal of Zoology 68, no. 8: 1645–1651.

[ece372905-bib-0059] Proctor, M. F. , C. T. Lamb , J. Boulanger , et al. 2023. “Berries and Bullets: Influence of Food and Mortality Risk on Grizzly Bears in British Columbia.” Wildlife Monographs 213, no. 1: e1078. 10.1002/wmon.1078.

[ece372905-bib-0060] Proctor, M. F. , B. N. McLellan , G. B. Stenhouse , G. Mowat , C. T. Lamb , and M. S. Boyce . 2020. “Effects of Roads and Motorized Human Access on Grizzly Bear Populations in British Columbia and Alberta, Canada.” Ursus 2019, no. 30e2: 16–39.

[ece372905-bib-0061] Proctor, M. F. , B. N. McLellan , C. Strobeck , and R. M. R. Barclay . 2004. “Gender‐Specific Dipsersal Distances of Grizzly Bears Estimated by Genetic Analysis.” Canadian Journal of Zoology 82, no. 7: 1108–1118.

[ece372905-bib-0062] Proctor, M. F. , B. N. McLellan , C. Strobeck , and R. M. R. Barclay . 2005. “Genetic Analysis Reveals Demographic Fragmentation of Grizzly Bears Yielding Vulnerably Small Populations.” Proceedings of the Royal Society B: Biological Sciences 272, no. 1579: 2409–2416. 10.1098/rspb.2005.3246 PMC1559960 16243699

[ece372905-bib-0063] Proctor, M. F. , S. E. Nielsen , W. F. Kasworm , et al. 2015. “Grizzly Bear Connectivity Mapping in the Canada–United States Trans‐Border Region.” Journal of Wildlife Management 79, no. 4: 544–558. 10.1002/jwmg.862.

[ece372905-bib-0064] Proctor, M. F. , D. Paetkau , B. N. McLellan , et al. 2012. “Population Fragmentation and Inter‐Ecosystem Movements of Grizzly Bears in Western Canada and the Northern United States.” Wildlife Monographs 180: 1–46. 10.1002/wmon.6.

[ece372905-bib-0065] Proctor, M. F. , C. Servheen , S. D. Miller , W. F. Kasworm , and W. L. Wakkinen . 2004. “A Comparative Analysis of Management Options for Grizzly Bear Conservation in the U.S.‐Canada Trans‐Border Area.” Ursus 15, no. 2: 145–160.

[ece372905-bib-0066] Radandt, T. G. 2009. “Recovery of Grizzly and American Black Bears From Xylazine, Zolazepam, and Tiletamine.” Ursus 20, no. 2: 114–119.

[ece372905-bib-0091] Ransom, J. I. , A. L. Lyons , K. C. Hegewisch , and M. Krosby . 2023. “An Integrated Modeling Approach for Considering Wildlife Reintroduction in the Face of Climate Uncertainty: A Case for the North Cascades Grizzly Bear.” Biological Conservation 279: 109947. 10.1016/j.biocon.2023.109947.

[ece372905-bib-0067] Richards, R. T. , and S. J. Alexander . 2006. A Social History of Wild Huckleberry Harvesting in the Pacific Northwest. (PNW‐GTR‐657; p. PNW‐GTR‐657). U.S. Department of Agriculture, Forest Service, Pacific Northwest Research Station. 10.2737/PNW-GTR-657.

[ece372905-bib-0068] Robbins, C. T. 1993. Wildlife Nutrition and Feeding. Academic Press.

[ece372905-bib-0069] Robbins, C. T. , J. K. Fortin , K. D. Rode , S. D. Farley , L. A. Shipley , and L. A. Felicetti . 2007. “Optimizing Protein Intake as a Foraging Strategy to Maximize Mass Gain in an Omnivore.” Oikos 116, no. 10: 1675–1682.

[ece372905-bib-0070] Roberts, D. R. , S. E. Nielsen , and G. B. Stenhouse . 2014. “Idiosyncratic Responses of Grizzly Bear Habitat to Climate Change Based on Projected Changes in Their Food Resources.” Ecological Applications 24, no. 5: 1144–1154. 10.1890/13-0829.1. 25154102

[ece372905-bib-0071] Rode, K. D. , and C. T. Robbins . 2000. “Why Bears Consume Mixed Diets During Fruit Abundance.” Canadian Journal of Zoology 78, no. 9: 1640–1645.

[ece372905-bib-0072] Rode, K. D. , C. T. Robbins , and L. A. Shipley . 2001. “Constraints on Herbivory by Grizzly Bears.” Oecologia 128, no. 1: 62–71. 28547091 10.1007/s004420100637

[ece372905-bib-0073] Rouse, W. , R. H. Haas , J. A. Schell , and D. W. Deering . 1974. Monitoring Vegetation Systems in the Great Plains With ERTS. Vol. 1, 309–317. NASA. Goddard Space Flight Center 3d ERTS‐1 Symposium.

[ece372905-bib-0074] Rupp, D. E. , J. T. Abatzoglou , and P. W. Mote . 2017. “Projections of 21st Century Climate of the Columbia River Basin.” Climate Dynamics 49, no. 5–6: 1783–1799. 10.1007/s00382-016-3418-7.

[ece372905-bib-0075] Schwartz, C. C. , and G. C. White . 2008. “Estimating Reproductive Rates for Female Bears: Proportions Versus Transition Probabilities.” Ursus 19, no. 1: 1–12.

[ece372905-bib-0076] Shores, C. R. , N. Mikle , and T. A. Graves . 2019. “Mapping a Keystone Shrub Species, Huckleberry (*Vaccinium membranaceum*), Using Seasonal Colour Change in the Rocky Mountains.” International Journal of Remote Sensing 40, no. 15: 5695–5715. 10.1080/01431161.2019.1580819.

[ece372905-bib-0077] Sorensen, A. , G. Stenhouse , M. Bourbonnais , and T. Nelson . 2015. “Effects of Habitat Quality and Anthropogenic Disturbance on Grizzly Bear (*Ursus arctos horribilis*) Home‐Range Fidelity.” Canadian Journal of Zoology 93, no. 11: 857–865.

[ece372905-bib-0078] Souliere, C. M. , S. C. P. Coogan , G. B. Stenhouse , and S. E. Nielsen . 2020. “Harvested Forests as a Surrogate to Wildfires in Relation to Grizzly Bear Food‐Supply in West‐Central Alberta.” Forest Ecology and Management 456: 117685. 10.1016/j.foreco.2019.117685.

[ece372905-bib-0079] Stelmock, J. J. , and F. C. Dean . 1986. “Brown Bear Activity and Habitat Use, Denali National Park—1980.” International Conference on Bear Research and Management 6: 155–167.

[ece372905-bib-0080] Stoneberg, R. P. , and C. J. Jonkel . 1966. “Age Determination of Black Bears by Cementum Layers.” Journal of Wildlife Management 30, no. 2: 411–414.

[ece372905-bib-0081] Teisberg, J. E. , S. D. Farley , O. L. Nelson , et al. 2014. “Immobilization of Grizzly Bears ( *Ursus arctos* ) With Dexmedetomidine, Tiletamine, and Zolazepam.” Journal of Wildlife Diseases 50, no. 1: 74–83. 10.7589/2012-11-273. 24171564

[ece372905-bib-0082] Turnock, M. F. 2024. Conservation Genetics and Population Viability Analysis of the Selkirk and Cabinet‐Yaak Grizzly Bear Populations. University of Idaho—College of Graduate Studies.

[ece372905-bib-0083] USFWS . 1993. Grizzly Bear Recovery Plan (p. 181). U.S. Fish and Wildlife Service. https://www.govinfo.gov/content/pkg/GOVPUB‐I49‐PURL‐LPS37437/pdf/GOVPUB‐I49‐PURL‐LPS37437.pdf.

[ece372905-bib-0084] Wakkinen, W. L. , and W. Kasworm . 1997. Grizzly Bear and Road Density Relationships in the Selkirk and Cabinet‐Yaak Recovery Zones. US Fish and Wildlife Service.

[ece372905-bib-0085] Wakkinen, W. L. , and W. F. Kasworm . 2004. “Demographics and Population Trends of Grizzly Bears in the Cabinet‐Yaak and Selkirk Ecosystems of British Columbia, Idaho, Montana, and Washington.” Ursus 15, no. 1: 65–75.

[ece372905-bib-0086] Welch, C. A. , J. Keay , K. C. Kendall , and C. T. Robbins . 1997. “Constraints on Frugivory by Bears.” Ecology 78, no. 4: 1105–1119.

[ece372905-bib-0087] Zedrosser, A. , B. Dahle , and J. E. Swenson . 2006. “Population Density and Food Conditions Determine Adult Female Body Size in Brown Bears.” Journal of Mammalogy 87, no. 3: 510–518.

[ece372905-bib-0088] Zedrosser, A. , O.‐G. Støen , S. Sæbø , and J. E. Swenson . 2007. “Should I Stay or Should I Go? Natal Dispersal in the Brown Bear.” Animal Behaviour 74, no. 3: 369–376.

