# Supplementary material for: Huckleberry Habitat and Its Influence on Two Small Populations of Grizzly Bears (Ursus arctos)
Source: Ecol Evol. 2026 Jan 19;16(1):e72905. doi: 10.1002/ece3.72905 (PMC12815594; doi:10.1002/ece3.72905)
Supplement: Supplementary file 1 — Data S1: ece372905‐sup‐0001‐Supinfo.docx. [file ECE3-16-e72905-s001.docx]

**SUPPLEMENTAL MATERIALS**

**Methods** – *identifying foraging locations*

We analyzed plant cover and berry presence data alongside expert observer opinion in 2019–2020, allowing a year of training. We first considered independent expert opinion as to whether a site was an important huckleberry feeding location or not. Biologists were allowed an intermediate category regarding whether the site was of foraging importance (i.e., “possible”). Twenty-seven percent of sites were assigned “possible”, 42 percent “yes”, and 31 percent “no”. We then performed logistic regression analyses using only biologist opinions of certainty as the binary response (i.e., “yes” or “no” classifications) and variables collected at ground-truthed locations as the predictor variables. We set a cutoff at 12 percent mean coverage, which aligns with a 50 percent chance a biologist would concur the site is an important feeding location for a female grizzly bear. Using plant canopy, and not berry presence or abundance, lessened observer bias introduced from interannual variation in fruit production at a site. Explicitly, the model attempts to predict locations of female presence where huckleberry canopy coverage is greater than 12 percent. In other words, given expert knowledge and opinion as well as historic presence of a female grizzly bear at training data sites, we inferred a female grizzly bear had a greater than average chance of using predicted locations, if available (i.e., site is within bear’s range).

**Methods** - *establishing cutoffs*

Establishing habitat value cutoffs of validated models takes care. We assessed all model fit statistics along the range of log-odds model values (Table 3). Diagnostic odds ratio at all proposed cutoffs was above 1.0, indicating positive model performance at all cutoffs. We settled on four cutoff values that represent varying but important levels of model prediction: 1) minimizing type II error (rule-in habitat though it might be wrong), 2) balancing true positives and true negatives, 3) balancing false positives and false negatives (recall vs. precision), and 4) optimizing total model accuracy and defining highest quality habitat (Table 3). Because point cutoffs represent points at which model statistics have maximum predictive quality of differing nature, we decided to bin our cutoffs such that each categorical bin represents the quality of model prediction, and thereby huckleberry habitat. Raster cells with empirical probabilities between 0.5 and 0.55 indicate predicted locations with higher sensitivity (Type II error > 0.05), but lower specificity. Predicted probabilities between 0.55 and 0.66 represent locations where specificity and sensitivity are balanced. Probabilities between 0.66 and 0.73 represent locations where false positives and false negatives are balanced (precision = recall), while above 0.73 represent locations where combined accuracy of the model is maximized, false positives are few, but the false negative rate is higher (prediction too tight). This last category would include the highest quality predicted huckleberry habitat. All log-odds values were transformed to their corresponding explicit likelihood that the cell contains ground-truthed huckleberry habitat (Fig. S2). Selected cutoffs fit appropriately within the distribution of model values (Fig. S5).

Table S1. List of model fit statistics used to determine overall model classification performance and best model value cutoffs to assess huckleberry habitat quality. TP = true positives; TN = true negatives; FP = false positives; FN = false negatives

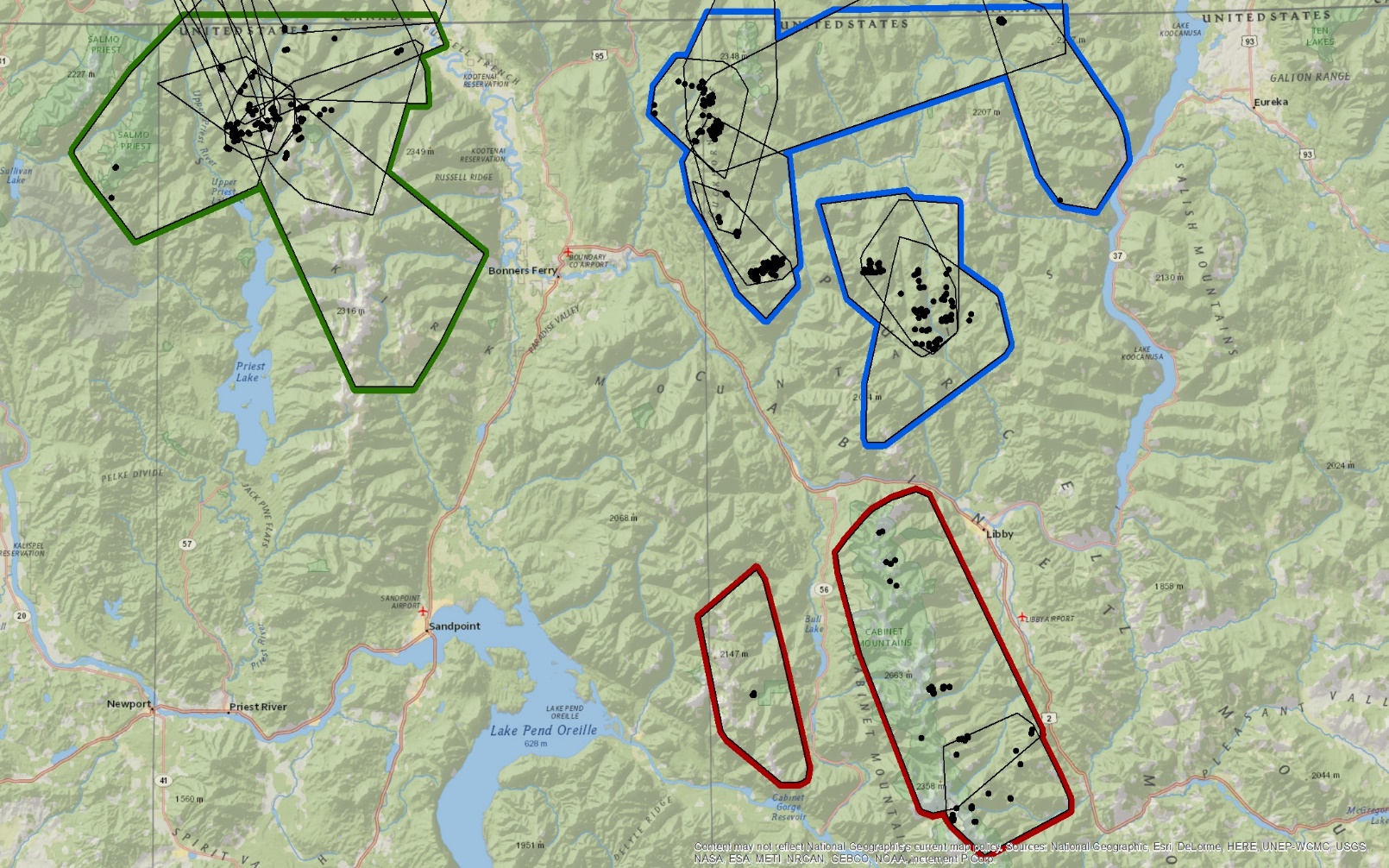


Figure S1. Map depicting defined available space in the Selkirk (green), Cabinet (red), and Yaak (blue) study areas. Thin black lines are individual female seasonal (July 15 – September 15) minimum convex polygon ranges, 2010–2019. Available space was defined as the composite minimum concave polygons of all sampled minimum convex female grizzly bear ranges within the United States. Black dots are ground-truthed female huckleberry feeding locations.


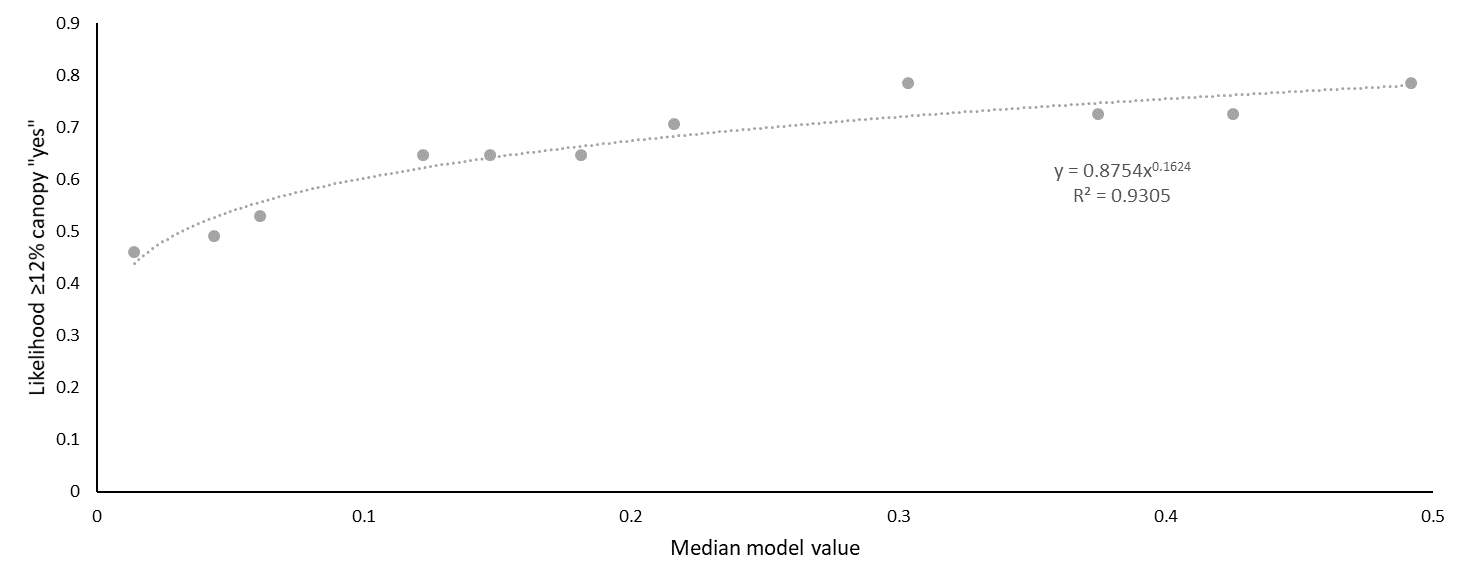


Figure S2. Fitted function used to transform the model *log-odds* output values at each predicted 100x100 meter raster cell to the explicit likelihood a cell contains female grizzly bear huckleberry habitat.

Figure S3. Mean huckleberry canopy coverage (percent) at female, berry season (July 15-September 15) GPS collar locations. At the same locations and using site context and expert knowledge, field biologists made an informed decision as to whether the visiting female grizzly bear was likely feeding on huckleberries at site (“1” on y-axis in graph; “yes”) or not (“0” on y-axis in graph; “no”). Red curve depicts the significant logistic regression curve (P < 0.001). At 12 percent canopy coverage, a biologist classifies a site as a huckleberry feeding location 50 percent of the time.

Figure S4. The thick blue line depicts the receiver operating characteristic (ROC) curve for Cabinet-Yaak-Selkirk female grizzly bear huckleberry habitat model. The 1:1 line defines a model with no predictive ability, a random classifier. Area Under the ROC Curve indicates a predictive model (AUC = 0.865). We selected model cutoffs at model values corresponding to the stars on graph. See text for more explanation.


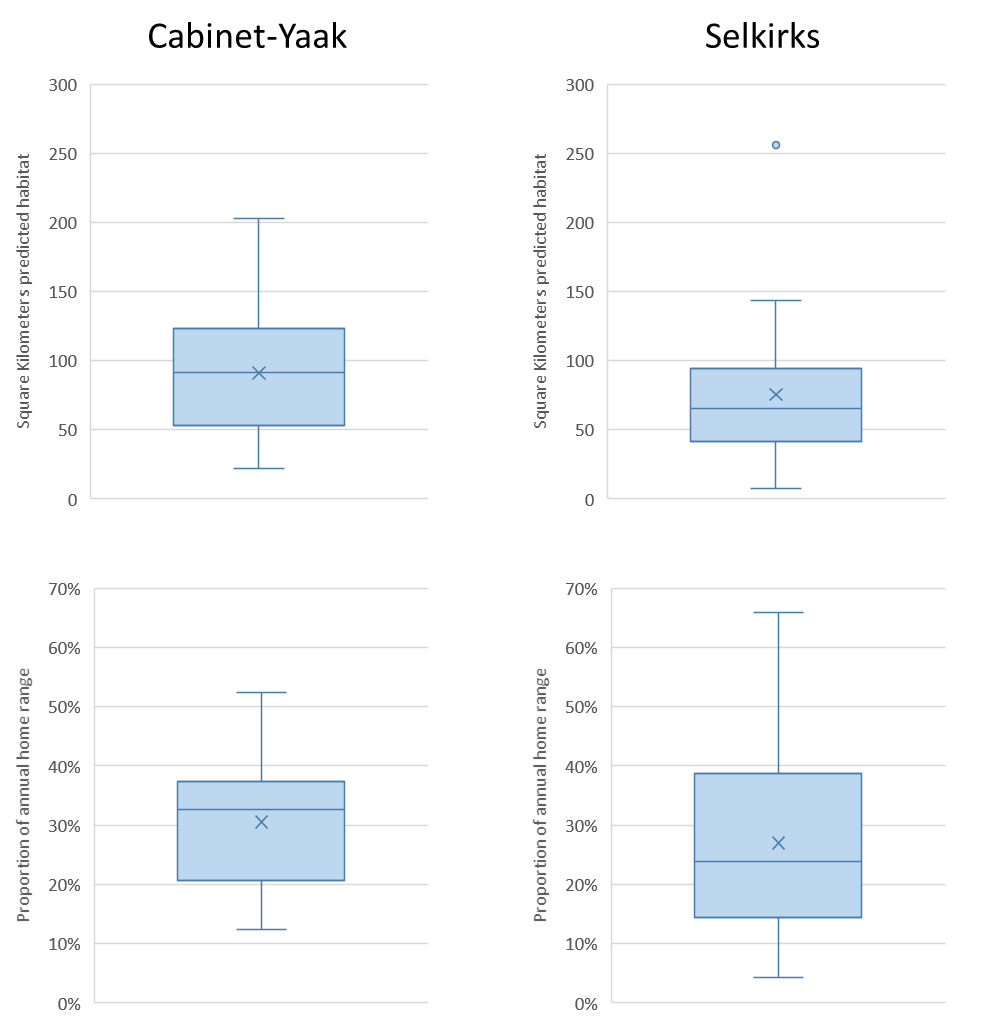


Figure S5. Box and whisker plots of area (km^2^) and proportion of adult female annual home ranges containing predicted huckleberry habitat in the Cabinet-Yaak and Selkirk Ecosystems (N = 36 and 31 bear-years, respectively), 2010–2021.


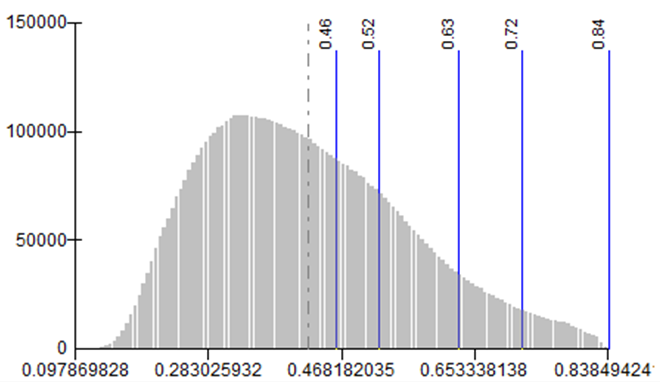

Figure S6. Distribution profile of predicted landscape at specified cutoffs (blue vertical lines, see Table 2). X-axis denotes the probability of a raster cell containing female grizzly bear productive huckleberry habitat. Y-axis is a count of binned values. Vertical dashed line is the mean probability value (0.42). SD = 0.14).


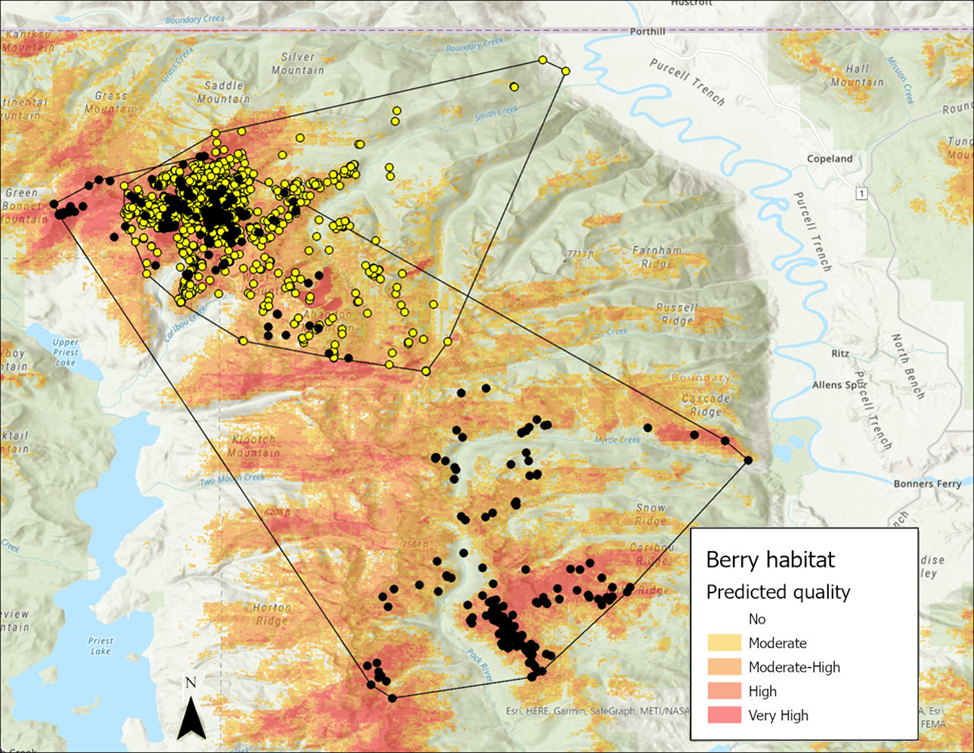


Figure S7. Seasonal locations (July 15 – September 15; prime huckleberry foraging) for known grizzly bear mother-offspring dyad of adult female #2003 (yellow) and subadult female #3021 (black), Selkirk mountains, northern Idaho, 2012–2013.


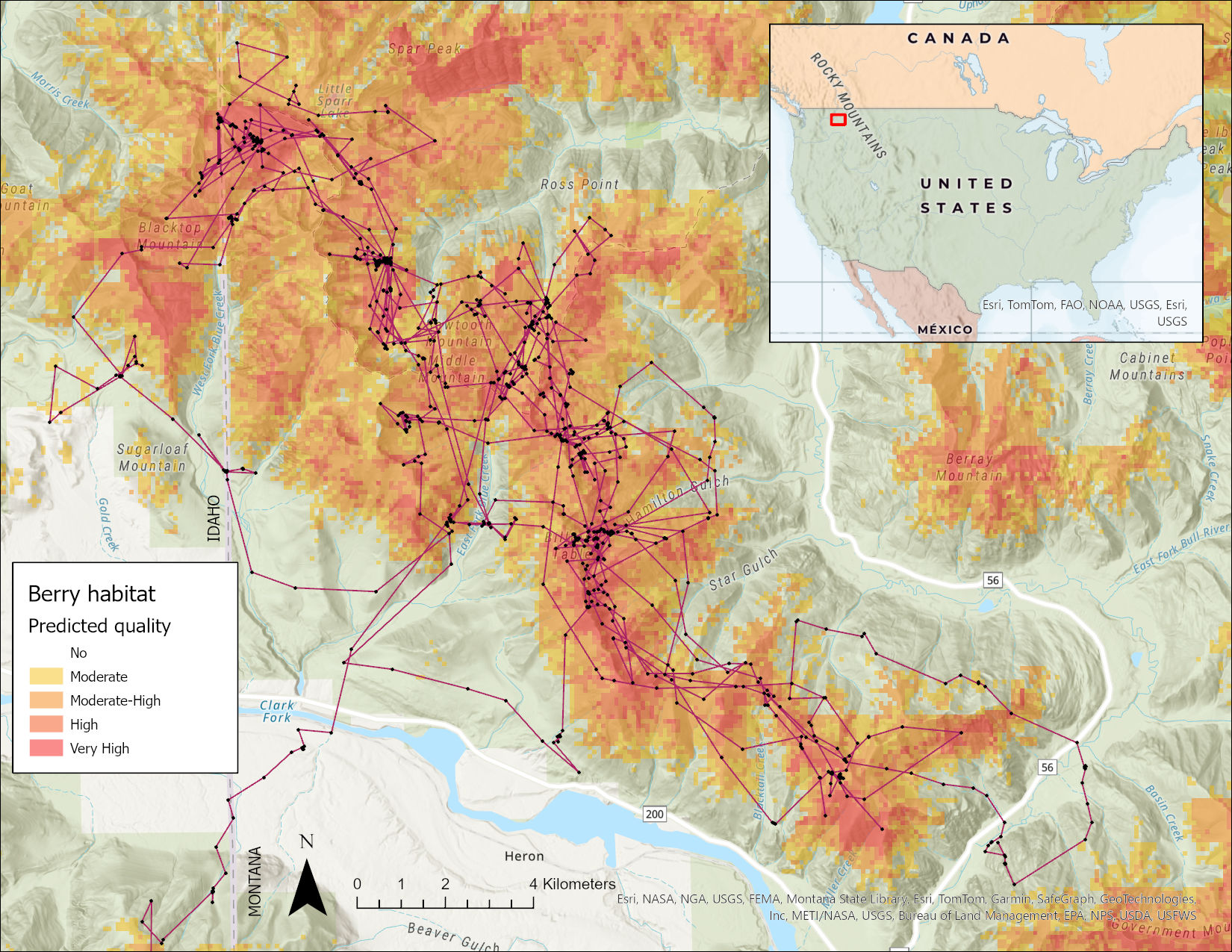


Figure S8. Locations and path of adult female grizzly bear 890 in the Cabinet Mountains of Montana and Idaho and across a state highway (MT 200), July–September 2022. High quality food habitat in proximity to major highways is important to consider in terms of connectivity and potential linkage areas.

**
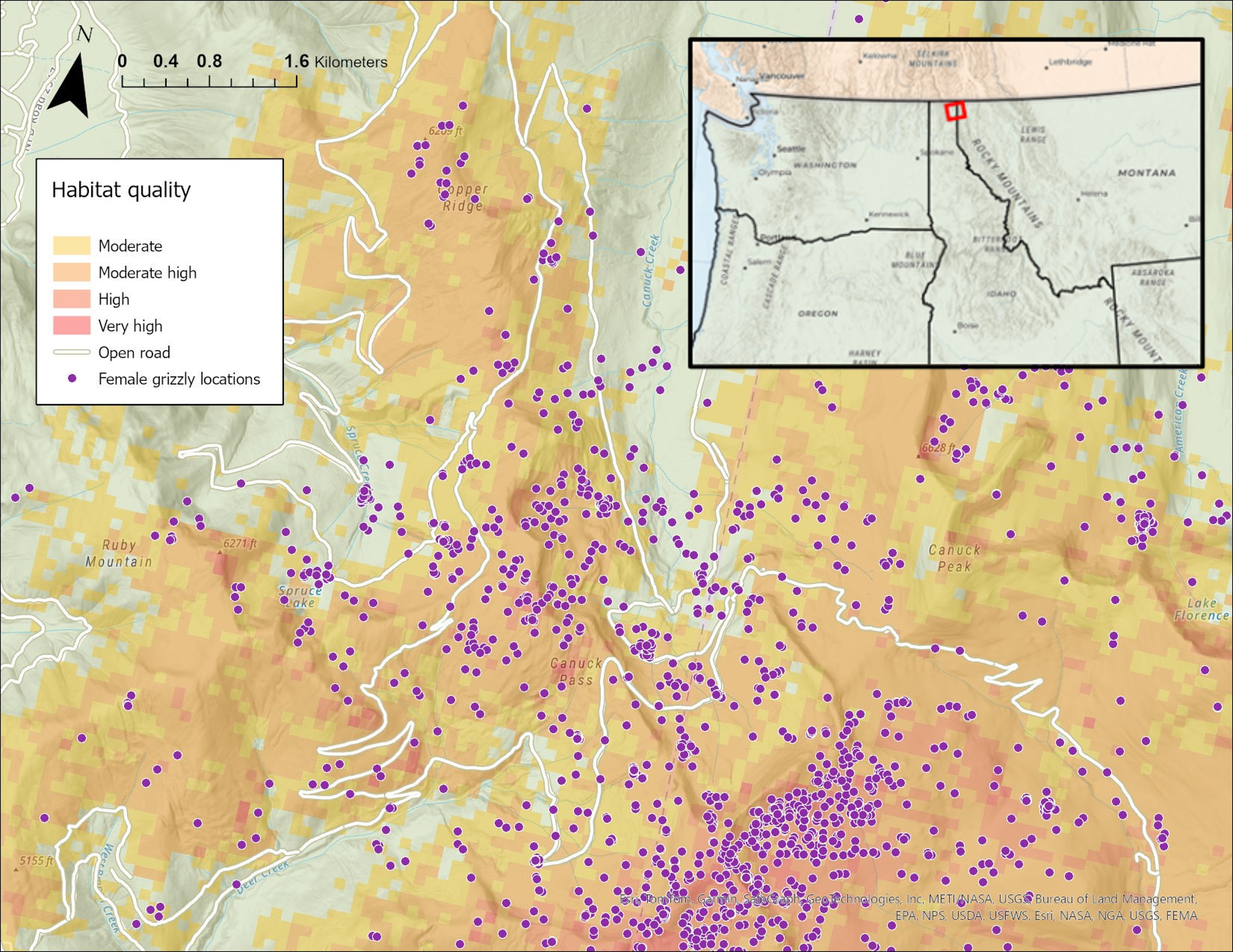
**

Figure S9. Example of an area where predicted huckleberry habitat may intersect open roads, Yaak study area, with relative quality of habitat included. Many human-caused mortalities to grizzly bears occur in proximity to open roads. Our bottom-up modeling approach identifies nutritionally important habitat regardless of mortality risk or displacement effects.


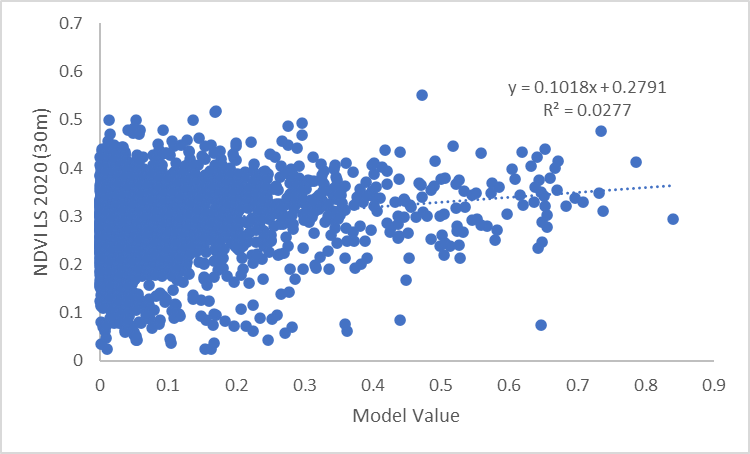


Figure S10. Relationship between predicted habitat model and NDVI values from July and August 2020.


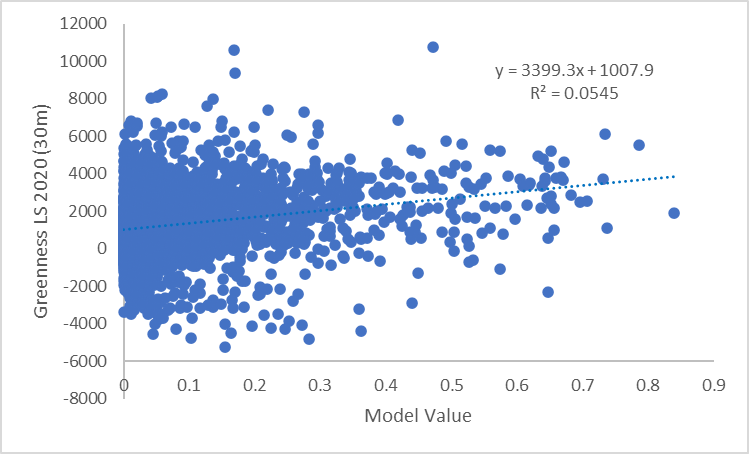


Figure S11. Relationship between predicted habitat model and remotely sensed greenness values from July and August 2020.
